# Seasonal dynamics of phytoplankton in the northern part of Suez Gulf, Egypt

**DOI:** 10.1007/s10661-023-11688-7

**Published:** 2023-08-17

**Authors:** Mostafa El-Sheekh, Mahmoud Abu-Faddan, Atef Abo-Shady, Mohamed Zein Alabdein Nassar, Wagdy Labib

**Affiliations:** 1 Botany Department, Faculty of Science, Tanta University, Tanta, Egypt; 2 National Institute of Oceanography and Fisheries (NIOF), Cairo, Egypt

**Keywords:** Phytoplankton dynamics, Environmental heterogeneity, Seasonal variation, The Suez Bay

## Abstract

This study was conducted to evaluate the seasonal variability of phytoplankton in the northern part of the Gulf of Suez (Suez Bay), considering the contribution of physicochemical parameters of bay water in shaping the dynamics, and eutrophication assessment. Water and phytoplankton samples were collected seasonally at nine stations in the Suez Bay during the period from the winter to autumn of 2012. A total of 423 phytoplankton species were identified, comprised mainly of 224 diatoms, 127 dinoflagellates, 33 cyanophytes, 20 chlorophytes, and 9 euglenophytes; the rest of the species (10 species) belong to other six groups. Of these, 28 species were potentially harmful. The total phytoplankton abundance exhibits a significant seasonal variation, with the autumn being the most fertile season, followed by the winter due to the proliferation of diatom species *Thalassionema nitzschioides* and *Proboscia alata* f*. gracillima*, respectively. While the seasonal species richness indicates that the winter attained the highest number of species, followed by summer. Generally, the major diatom genera were *Chaetoceros* (16 species), *Navicula* (15 species), *Nitzschia* (15 species), and *Amphora* (14 species), while dinoflagellates were principally composed of the genera *Protoperidinium* (34 species), and *Tripos* (26 species). Water temperature, pH, salinity, nitrate, and nitrite were the most important explanatory parameters in regard to phytoplankton abundance and chlorophyll *a* concentration. In addition, the phytoplankton stability exhibited a significant positive relationship with the mean values of dissolved oxygen and biological oxygen demand and the variability of salinity and phosphate, while a negative relationship was observed with ammonia and nitrite and the variability of nitrate. Based on the trophic index (TRIX), the bay water was classified as mesotrophic (moderately polluted) for almost the entire year except in the autumn as it turned eutrophic. The results explored the potential importance of the environmental heterogeneity in the bay as a key structuring mechanism of phytoplankton abundance and biomass, influenced by anthropogenic activities.

**Supplementary Information:**

The online version contains supplementary material available at 10.1007/s10661-023-11688-7.

## Introduction

Phytoplankton merits special attention for being generally the dominant primary producer in most aquatic ecosystems, responsible for about half of the world’s primary production, and thus a key player in the global carbon cycle (Boyce et al., 2010). Such high productivity is promoted by Fast turnover rates of phytoplankton (often a few days) in response to dynamic surrounding environmental conditions (Schabhüttl et al., 2013). They are also fundamental in the trophic energy transfer of nearly all marine ecosystems, from microscopic zooplankton to whales, and have key roles in carbon sequestration (Basu &Mackey, 2018), oxygen production (Falkowski, 2012), as well as controlling the biogeochemical cycles in aquatic environments (Sigman & Hain, 2012). All changes in phytoplankton composition, abundance, and distribution may have cascading effects along the trophic food web (Lehtinen et al., 2016). The spatiotemporal distribution of phytoplankton assemblages can be highly affected by physical, chemical, and biological processes (Macintyre & Cullen, 1996). Phytoplankton growth depends on the availability of nutrients such as nitrate, phosphate, and silicate at various levels depending on the species (Sigman & Hain, 2012). Phytoplankton species have shorter generation times, are more directly responsive than others to minor environmental oscillations on a shorter temporal scale and have a high sensitivity to instantaneous abiotic and biotic variations (Cloern & Dufford, 2005). The information gained on the phytoplankton standing crop, relative diversity, and taxonomic richness of its community are useful tools to monitor changes in the state of coastal marine environments, and to appraise the effectiveness of integrated coastal management (Loreau, 2010).

The Suez Bay waters are a hydro-dynamically complex system that undergoes intense and continuous environmental pressure derived from various types of pollution, including sewage, agricultural products, industrial effluents, organic compounds, plastics, thermal pollution, ship-based sources, and biological pollution or bioinvasion (Nour et al., 2022). Phytoplankton responds to different types of pollution not only through changes in abundance but also through species dominance and succession patterns and community structure as species diversity, evenness, and richness, which leads to a food shortage at the next trophic levels: micro- and mesozooplankton (Rothenberger et al., 2013).

Historically, knowledge of the Red Sea phytoplankton is derived mainly from Halim’s review in (1969). Since then, numerous studies have been conducted in the Suez Bay and adjacent waters. Nassar and Hamed (2003) recorded 80 species and varieties, with two main phytoplankton peaks in the spring and autumn under high levels of nutrients. Nassar (2007) explained that, in comparing his work at different locations along the western coast of the Suez Gulf with his previous data, many aspects had changed, such as increased species diversity and standing crop, common dominance of diatoms, active sharing of several newly recorded Chlorophyta species that seem to be affected by discharged freshwater input, the main productive period between autumn and winter, and distinct regional distribution. Madkour et al. (2010) found that the phytoplankton population along the Egyptian coastal regions of the Red Sea was diversified (181 species) and comprised mainly dinoflagellates (116 species) and, to a much lesser extent, diatoms (60 species). Nassar et al. (2014) reported additional evidence for high productivity during autumn in the coastal waters of the northern part of the Red Sea, and diatoms, as usual, were the main contributor (76.4% of the total), followed by dinoflagellates (14.63%). Nassar and Khairy (2014) offered a checklist of phytoplankton species in the Egyptian waters of the Red Sea and some surrounding habitats found between 1990 and 2010. They reported 207 species, including Bacillariophyceae (116 species, 15 genera) and Dinophyceae (48 species, 11 genera). Nassar et al. (2015), based on seasonal collection from the eastern coast of the Suez Gulf during autumn 2012 and winter, spring, and summer 2013, reported a diversified phytoplankton community with 138 species. Ismael (2015) reported that the phytoplankton of the entire Red Sea comprises 389 species and varieties, an increase of 181 species since Halim’s review in 1969. Gittings et al. (2018) reported that the northern Red Sea is characterized by a distinct winter phytoplankton bloom, while warmer stratified conditions in the summer contribute to less vertical mixing and a reduction in phytoplankton abundance. Recently, Nassar and Fahmy (2023) studied the seasonal variability of phytoplankton along some of the Red Sea harbors. The obtained results revealed 119 phytoplankton species, including 80 species of diatoms, 27 species of dinoflagellates, and six species of both cyanophytes and chlorophytes.

The main goal of this research is to evaluate the spatiotemporal variability of phytoplankton in the Suez Bay over a year-cycle survey, considering the contribution of physical and chemical interactions in shaping the dynamics, and eutrophication assessment. An understanding of the synergistic interactions between the different ecological aspects, such as eutrophication and phytoplankton dynamics, will be useful to devise suitable remediation strategies for future research.

## Materials and methods

### Study area and chosen stations

Suez Bay is a shallow extension of the Gulf of Suez, which is a large, semi-closed area, roughly elliptic in shape, located between longitudes 32º 28 `25 and 32º 34 `32 E and latitudes 29º 54 `and 29º 57 `N. The average length along the major axis is 13.2 km, and the average width along the minor axis is 8.8 km, while its surface area is 77.13 km^2^. The bay is connected to the Gulf of Suez through most of its southeastern side and connected to the Suez Canal from the northern terminus. The water from the Suez Gulf enters the bay from the eastern side (Sinai side) and discharges from the western side. Therefore, there is a persistent anticlockwise circulation in the bay, which enhances the pollution on the western side rather than the eastern one (Hamed et al., 2010). In addition, the water current in the Suez Canal generally flows from southward (Suez Bay) to northward directions, except in the summer (July, August, and September), when it is reversed to the south (Morcos, 1960), enhancing the Lessepsian and anti-Lessepsian migration of phytoplankton species, respectively (Por, 2012). The city of Suez and its major industries occupy the northern part of the Bay.

### Collection of samples

**Fig. 1:**
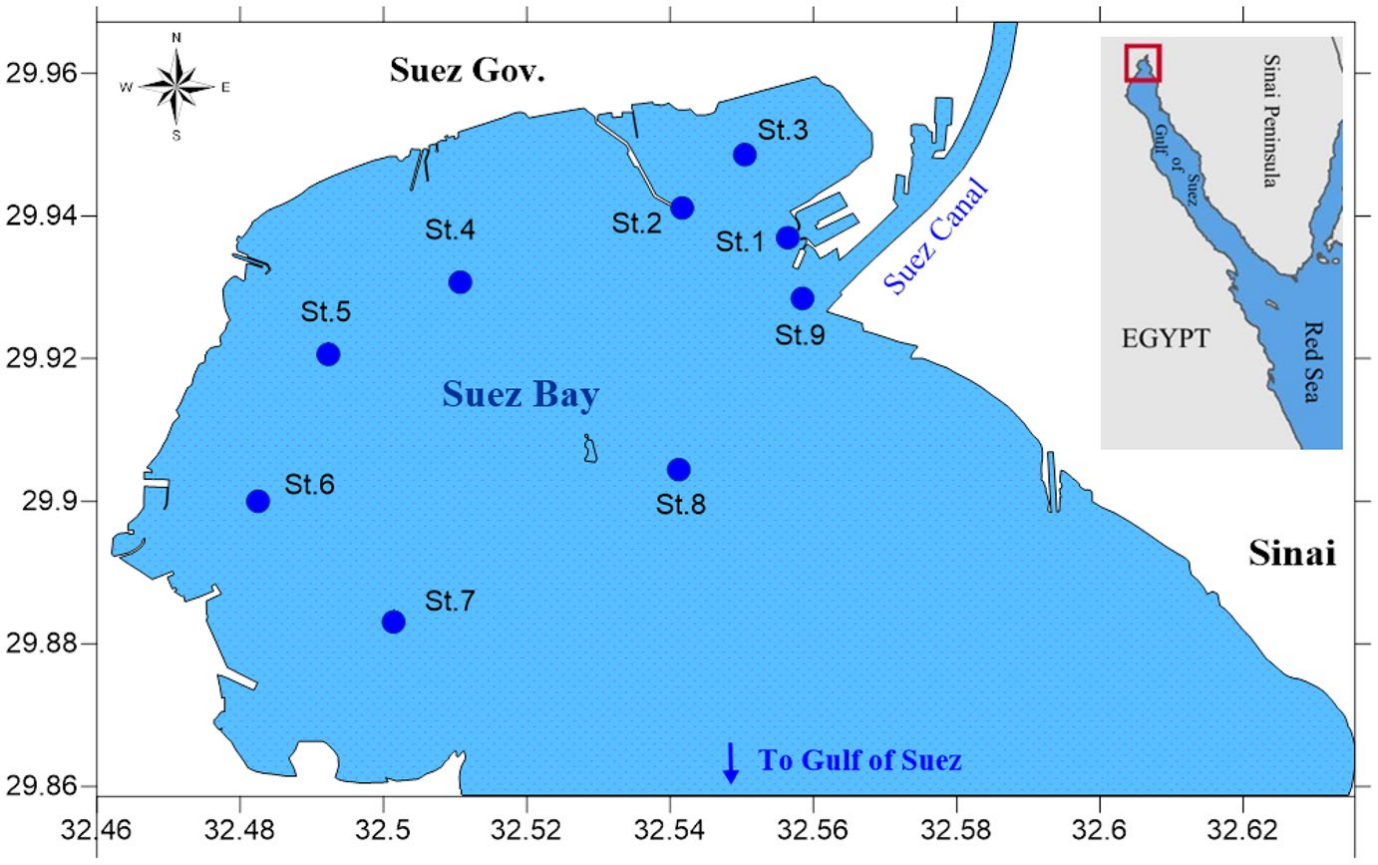
Map of Suez Bay and sampling locations

Water and phytoplankton samples were collected from nine stations in the Suez Bay during a year cycle as represented by February 2012 (winter), May (spring), August (summer), and November (autumn). The selection of these stations took into consideration the coverage of the entire study area and representation of all existing marine activities and types of pollution (i.e. Sts.1 &9; ship-based activities, Sts. 2&3; petroleum oil, St4; industrial and sanitary drain, St5; thermal pollution and sewage, St6; ship building industry, St7; ship-based activities, industrial drain, and sewage, St8; relatively far from any pollution source, located towards the east of the bay was assumed reference station) (Fig. 1).

### Physicochemical analysis

Surface water samples were collected using a water sampler for chemical investigation. Dissolved oxygen and ammonia were fixed instantly by the addition of specified reagents to their respective collection bottles (Strickland & Parsons, 1972). Samples for nutrient and chl. *a* analysis were collected in polyethylene bottles of 2 liter capacity, except for inorganic phosphate, which was stored in a hard glass bottle. The water samples were kept directly in an ice box (at <8 °C).

Temperature (^o^C) and pH values of seawater were measured in the field immediately after sampling using a pocket pH meter (model Orion 210), and salinity was measured using a Beckman (No. R.S.7C) salinometer. Transparency (m) was measured using Secchi disk according to the methods of Rogers et al. (1994). The concentrations of silicate (SiO_4_), nitrate (NO_3_), nitrite (NO_2_), dissolved inorganic phosphate (PO_4_), ammonia (NH_4_), and dissolved oxygen (DO) were determined using Strickland and Parsons (1972) method. The biological oxygen demand (BOD) was calculated by subtracting the dissolved oxygen fixed after five days of storing a seawater sample in the dark at room temperature from the value of dissolved oxygen fixed at the time of sampling. Chemical oxygen demand (COD) was estimated according to Ellis et al. (1946). The molar concentrations of each nutrient (NH_4_, NO_3_, NO_2_, PO_4_, SiO_4_) were used to determine molar ratios for total nitrogen (N): total phosphorus (P) and silicon (Si): total nitrogen (N), and were extrapolated for the N : P : Si Redfield ratio as 16 : 1 : 15 (Redfield, 1958) to predict nutrient limitation (Lv et al., 2011); limited by N when N:P<10, co-limited by N and P when 10<N:P<17, limited by P when N:P>17, and limited by Si when Si:N>15:16.

### Phytoplankton sampling and analysis

By means of a specific phytoplankton net, with a 20 μm mesh and an upper diameter of 40 cm, samples were collected following the vertical hauls method, according to APHA Standard Methods for the Examination of Water and Wastewater (10200 plankton) (APHA, 1995). The Taxonomic identification was mainly performed according to Taylor (1976) and Tomas (1997). All taxa have been verified as accepted species and given the currently accepted name as defined by the World Register of Marine Species database (WoRMS, http://www.marinespecies.org/aphia.php?p = web service) and Algae Base (http://www.algaebase.org/). Determination of abundance was performed by means of the Sedgewick-Rafter slide according to APHA (1995).

Chlorophyll *a* was determined according to the methods of Strickland and Parsons (1972). The temporal stability index (TSI) was calculated as the variation coefficient of phytoplankton biomass using both the temporal mean values and the variability, measured as standard deviation, of chlorophyll *a* according to Tilman et al. (2006). The trophic index (TRIX) was calculated for each station according to Pavlidou et al. (2015).

### Phytoplankton diversity

A set of ecological indices, including the number of phytoplankton species (*S*); total number of individuals (*N*); Shannon’s Diversity Index (*H'*; log e base) (Shannon & Weaver, 1949), and the Evenness Index (*J*') (Pielou, 1966), were applied to describe the alpha diversity. In addition, species turnover across time (temporal β diversity) in Suez Bay is measured by Whittaker's species turnover (βW) (Whittaker, 1972) and Beta turnover (βT) (Cody, 1975).

### Data analysis

Pearson’s simple correlation was applied to analyze the relationships between environmental factors and different biological variables. Multivariate analyses were used to identify environmental parameters that affect phytoplankton community structure. Prior to multivariate analyses, species abundances were square-rooted. The biological similarity matrices were constructed using the Bray-Curtis index. The seasonal variability of phytoplankton assemblages was formally examined using per-mutational multivariate analysis of variance (PERMANOVA) and analysis of similarities (ANOSIM). Then, Principal coordinate analysis (PCO) coupled with cluster analysis was performed. These were performed using PRIMER 6 (6.1.16). In addition, the principal components analysis (PCA) was performed using TSI of the nine sampling sites and nutrient variables and SD of nutrient variables. In order to visualize the spatial distribution pattern of Chl. *a* and Shannon’s Diversity Index among different stations, 2D contour Grid-based maps, applying the krigging method for gridding, were performed in Software Surfer version 13.0.383.

## Results and discussion

### Environmental characteristics of seawater

#### Physicochemical parameters

The surface water temperature oscillated normally between 19.1 ºC in the cold winter (St. 2) and the highest of 34.4 ºC in the summer (St. 5), reflecting marked seasonality. The spring warming observed was accompanied by a rise in temperature of 4-5 ºC, compared with the winter. The surface water salinity fluctuated within a wide range of variation, hardly decreased below 40 psu (39.7 psu, spring, St. 8), and reached its highest level of 45.4 psu in the summer, St. 1, with an annual mean of 42.68 psu. (See Appendix 1, Table 1). Generally, salinity in the Gulf of Suez and Suez Bay is comparatively very high as 40-42.4 psu (Alraddadi, 2013), which seems compatible with the present record. The annual mean of water transparency (3.2 m) reflects a wide range between 2 m (spring, St. 9, and autumn, St. 3), and the highest of 5.5 m in the winter, St. 8. Based on the mean values, a limited variation was observed in the spring and winter (3.1 ± 0.32 m and 3.32 ± 0.39 m, respectively).

The measured pH values lie in the alkaline range, varying slightly within 0.81 units between 7.79 (winter, St. 3) and 8.6 (autumn, St. 4), with an annual mean of 8.27. However, this difference in pH might affect the phytoplankton community structure. Fabry et al. (2008) said a decrease from normal pH of 0.5 units or less appears to be tolerated well by most organisms, whereas a decrease of 0.5–1 units or more can result in stress responses and more serious deleterious effects. Generally, the productivity and viability of aquatic organisms are better when the pH of the surrounding environment is maintained as constant rather than undergoing large variations (Harding, 1992). Dissolved oxygen concentrations never fell below 3.25 mg l^-1^ (winter, St. 6) and peaked at 7.3 mg l^-1^, autumn, St. 1 (annual average 5.2 mg l^-1^). Dissolved oxygen is used as an indicator in most water quality studies (U.S. EPA, 2001). The investigated stations experienced DO >3<4 mg l^-1^ by about 17% of the total oxygen measurements and DO >4<5 mg l^-1^ by almost the same percentage. There were no situations of the criteria for hypoxia in the bay; hypoxia is the situation in which the dissolved oxygen falls to values less than 2 mg l^-1^ or slightly higher (Bianchi et al., 2010) as the limit of survival. Sheldon and Alber (2011) classified the water types based on DO criteria as 3 and 5.5 mg l^-1^ the "fair/poor" and "good/fair" boundaries, respectively. According to Gray et al. (2002), growth is affected by dissolved oxygen between 4.5 and 6.0 mg l^-1^, and aspects of metabolism are affected between 2 and 4 mg l^-1^. The oxygen saturation determined by means of salinity and temperature showed that most of the sampling locations were less than 100% saturation. The values of BOD explain a distinct difference between a minimum of 0.33 mg l^-1^ (spring, St. 9) and a maximum of 5.36 mg l^-1^ (autumn, St. 1), with an annual mean of 1.81 mg l^-1^. Except for the high values observed in the autumn, accompanied by the phytoplankton overgrowth, the BOD concentrations were low; they never exceeded 2.93 mg l^-1^. The values above the critical level (2 mg l^-1^) contributed to 27.7% of the total measurements, giving caution for ongoing rises in progressive eutrophication processes (Verity et al., 2006). The values of chemical oxygen demand** (**COD) varied from a minimum of 7.2 mg l^-1^ (winter, St. 8) to its extreme of 26.4 mg l^-1^ (winter, St. 2), with an annual mean of 1.8 mg l^-1^. COD concentrations were lowest at St. 4 and 8, and highest at St. 2, which was distinguished by ship arrival and departure and specialized in petroleum oil transportation. The dissolved ammonia concentrations contributed significantly to the total nitrogen (63.46%) and showed a distinct seasonal variation from a minimum of 3.7 µg l^-1^ in the summer, St. 4 to a maximum of 41.38 µg l^-1^, spring, St. 5, with an annual mean of 12.6 µg l^**-1**^. The dissolved nitrite concentrations (Annual mean; 1.9 µg l^-1^) were always low, ranging from 1.083 µg l^-1^ in the autumn, St. 9 to 2.93 µg l^-1^ in the summer, St. 7. The dissolved nitrate concentrations varied dramatically between the exhausted value at 0.40 µg l^-1^ in the spring, St. 3 and its extreme of 18.47 µg l^-1^ in the autumn, St. 3, with an annual mean of 5.36 µg l^-1^. The phosphate concentration was at a minimum of 1.47 µg l^-1^ in the spring at St. 6) and increased by about 7-fold in the autumn at St. 1 (11.22 µg l^-1)^, attaining an annual mean of 3 µg l^-1^. Dissolved silicate concentrations varied significantly between 1.55 µg l^-1^ (autumn, St. 4) and 14.05 µg l^-1^ (spring, St. 1), with an annual mean of 5.87 µg l^-1^**.** The mean seasonal concentration of key eutrophication elements in the bay, i.e., total phosphorous (3.13 ± 0.7 µg l^-1^), total nitrogen (19.92 ±2.84 µg l^-1^), and silicate (5.87±0.98 µg l^-1^) substantially exceeded the previous record of Nassar and Hamed (2003) and Shams El-Din et al. (2005), indicating increased eutrophication is on the rise (see Appendix 1, Table 1). In general, Variation in the water temperature may be mainly due to the effect of a season (Jayaraman et al., 2003), while salinity, pH variations, and nutrients variability have to follow uncertain volumes of discharged effluents from different land-based sources (Snedden et al., 2007).

#### Nutrient ratios

The results showed that 75% of stations were limited by total phosphorus, As per the findings limitation by phosphorous (N/P > 20) was the major, representing 75% of stations, while 8.33% of stations were limited by total nitrogen (N/P < 10) and 16.66% co-limited by N and P (10 <N/P < 20). In addition, all stations were limited by silicate (Si/N < 15:16). The current changed N/P ratio, which is prone to eutrophication (Luo et al., 2007), represent a challenge to the rigid N:P ratio of 16:1 (Redfield, 1958), which was considered a benchmark to differentiate N limitation and P limitation and as a reference point for the upper limit of N:P in seawaters (Lenton & Watson, 2000) (See Appendix 1, Table 2).

#### Phytoplankton community structure

The phytoplankton community is characterized by 423 taxa, overwhelmingly dominated by diatoms (224 species), followed by dinoflagellates (127 species), and to a much lesser extent by Cyanophyta, Chlorophyta, and Euglenophyta (33, 20, and 9 taxa, respectively). Other recorded taxonomic groups (Raphidophytes, Eustigmatophytes, Charophytes, Silicoflagellates, Haptophytes, Ebriids) of rare occurrences are represented by one to a few taxa. The major diatom genera were *Chaetoceros* (16 species), *Navicula* (15 species), *Nitzschia* (15 species), *Amphora* (14 species), and *Pleurosigma* (11 species), While dinoflagellates were principally composed of the genera *Protoperidinium* (34 species), *Tripos* (26 species) and *Prorocentrum* (10 species) (See Appendix 1, Table 3). This species richness is significantly higher than the records of Deyab et al. (2004), Nassar (2007), Nassar and Khairy (2014), and Madkour et al. (2010) at the northern coast of the Suez Gulf and adjacent areas. Such increased dissimilarity over long time intervals is attributable to increasing environmental variability through time (Halley, 1996), and different populations respond differently to environmental fluctuations (Schwaderer et al., 2011).

## Seasonal variation of phytoplankton assemblages

### Winter

The community is composed of 278 species. The assemblage was classified as 155 diatom species (84.46% of the total species number), with an average of 27710 cells l^-1^, 88 dinoflagellate species (14.33%, 4700 cells l^-1^), 17 Cyanophyta species (0.74%, 243 cells l^-1^), 9 Chlorophyta species (0.25%, 83 cells l^-1^), 6 Euglenophyta species (0.17%, 65 cells l^-1^), and one species each Raphidophyta, Eustigmatophyta and Haptophyta that form together about 0.06 %, with an average of 6 cells l^-1^ for each (Table 1). The most dominant species during winter was *Proboscia alata* f*. gracillima* (64.6 %, 21200 cells l^-1^). Other major diatom genera: *Nitzschia* (12 species), *Chaetoceros* (11 species), *Navicula* (10 species), and *Pleurosigma* (10 species) in combinations, accounted for 5 % of the total mean diatom counts. The dinoflagellate genera *Tripos* (25 species) and *Protoperidinium* (20 species) were the main constituents (56 %). The regional distribution of the phytoplankton abundance shows the lowest mean at St. 8 (19385 cells l^-1^), while the highest is at St. 4 (39755 cells l^-1^).

**Table 1:** Seasonal phytoplankton community structure in Suez Bay

	**Winter**			**Spring**			**Summer**			**Autumn**		
	**S**	**N (Mean )**		**S**	**N (Mean )**		**S**	**N (Mean )**		**S**	**N (Mean )**	
		**cells l**^**-1**^	**%**		**cells l**^**-1**^	**%**		**cells l**^**-1**^	**%**		**cells l**^**-1**^	**%**
**Diatoms**	155	27708	84.46	77	7839	40.37	108	14933	94.99	107	69617	98.836
**Dinoflagellates**	88	4700	14.33	52	9711	50.01	65	612	3.89	50	687	0.975
**Cyanophytes**	17	243	0.74	10	72	0.37	11	54	0.35	9	62	0.087
**Chlorophytes**	9	83	0.25	7	1039	5.35	5	22	0.14	6	17	0.025
**Euglenophytes**	6	56	0.17	5	744	3.83	3	7	0.05	2	5	0.007
**Raphidophytes**	1	6	0.02	—	—	—	1	5	0.03	1	5	0.007
**Eustigmatophytes**	1	6	0.02	—	—	—	—	—	—	1	12	0.018
**Charophytes**	—	—	—	—	—	—	—	—	—	1	2	0.004
**Silicoflagellates**	—	—	—	1	11	0.06	1	81	0.52	1	27	0.039
**Coccolithophorids**	1	6	0.02	—	—	—	1	2	0.02	1	2	0.004
**Ebriids**	—	—	—	—	—	—	1	2	0.02	—	—	—
**Total**	278	32806		152	19417		196	15721		179	70437	

### Spring

The highest relative abundance of diatoms in the winter was overtaken by dinoflagellates in the spring. Among the community of 152 identified taxa, 52 dinoflagellate species representing 50 % of the total phytoplankton density (with an average of 9710 cells l^-1^) were the major constituent of the community, followed by diatoms (77 species, 40.37 %, average 7839 cells l^-1^), 10 cyanophytes (0.37 %, 72 cells l^-1^), 7 chlorophytes (5.35 %, 1039 cells l^-1^), 5 euglenophytes (3.83%, 744 cells l^-1^), and one silicoflagellate (Table 1). Again, *Proboscia alata* f. *gracillima* predominated (4017 cells l^-1^), and *P*. *gracile* ranked second (2556 cells l^-1^), both, in combination, formed 33.8 % of the total mean phytoplankton counts. Other major diatom genera; *Pleurosigma* (8 species) and *Diploneis* (7 species) contributed together 8.5 % of the total mean diatom abundance, while the common genera *Protoperidinium* (19 species), *Tripos* (8 species), and *Prorocentrum* (8 species) prevailed and accounted for 70.7% of the total mean dinoflagellate abundances. The density (average value) ranged between 14450 cells l^-1^, St. 8, and the highest of 24675 cells l^-1^ in each St. 5 and 7.

### Summer

The phytoplankton community structure consisted of 196 species. Diatoms regained the highest degree of dominance in species diversity and abundance (108 species, 95 %, 14935 cells l^-1^), and dinoflagellates became of much less importance (65 species, 3.89%, 612 cells l^-1^). The other groups, cyanophytes (11 species), chlorophytes (5 species), euglenophytes (3 species), and one species each of raphidophytes, silicoflagellates, Haptophytes, and ebriids, made a negligible contribution (Table 1). The community was overwhelmingly dominated by *Thalassionema nitzschioides* (89.4%, 14057 cells l^-1^). The genera, *Amphora*, *Chaetoceros,* and *Navicula* (7 species for each) were of common occurrence at all stations. The major dinoflagellate genera were *Protoperidinium* (17 species) and *Tripos* (16 species), which together formed 44 % of the total mean dinoflagellate counts. The regional distribution declares a high population size at St. 7 (average 20111 cells l^-1^), and it was at the lowest at St. 8 (9532 cells l^-1^).

### Autumn

A total of 179 species were identified. Diatoms contributed almost the whole community (179 species, 98.84 % of the mean total counts, and an average of 69615 cells l^-1^), while dinoflagellates comprised 0.98 % and 685 cells l^-1^. Others, including cyanophytes (9 species), chlorophytes (6 species), euglenophytes (2 species), and one species from each of the other groups, were of insignificant sharing (Table 1). The diatom species *Thalassionema nitzschioides* was still the leader (65430 cells l^-1^), and it was also followed by *Thalassionema frauenfeldii* (2865 cells l^-1^), both of which together formed 96.9 % of the total mean phytoplankton density. Other species of much less importance belong to the genera, *Nitzschia* (8 species), *Chaetoceros*, *Navicula,* and *Pleurosigma* (7 species for each). The dinoflagellates comprised the same previously reported genera as in the summer but with different species numbers: *Protoperidinium* (18 species), *Prorocentrum* (7 species), and *Tripos* (7 species). Considering the relatively high density in the autumn, the surface distribution shows St. 2 to have the highest abundance (84265 cells l^-1^), while St. 8 sustains the lowest (33220 cells l^-1^).

In general, the results declare that sampling sites had more similar species compositions than sampling dates; seasonality is more effective on community structure than regionality. In addition, significant differences in the relative contributions of the two major functional groups, diatoms followed by dinoflagellates, to the phytoplankton community and standing stock were noticed. Diatoms were the major constituent in the winter with a relative abundance of 84.46%, while dinoflagellates contributed 14.33%. In the spring, the relative sharing explained the significance of dinoflagellates (50.01%) and again, diatoms were almost the solo constituent (94.99-98.84%) in the summer and autumn. The diatoms: dinoflagellates ratio of species richness fluctuated between 1.4 and 2, confirming common diatom predominance, as previously reported by Nassar and Hamed (2003) and Madkour et al. (2010). Reasons for the diatoms dominance might be attributed to their wide tolerance towards vastly changing environmental conditions in the bay, similar to elsewhere (Weilhoefer & Pan, 2006). Among the large number of species recorded, the high degree of diatom species dominance was of paramount importance to the bay community. The ratio of centric to pennate diatoms based on the species number indicates the increased number of pennate species over the centric ones. This is probably accelerated by factors such as water mixing enhancement from active ship movement relative to the shallowness of some sampling stations. Based on the species diversity, the pennate diatoms contributed 52.9% in the winter to 71.4% in the summer, a greater abundance by about a 6.1-fold increase in the first season compared with the centric diatoms. The observed high frequency of pennate diatoms indicates a decrease in water clarity, in accordance with Cooper and Brush (1993). In addition, the proliferation of the pennate diatom *Thalassionema nitzschioides* raised the relative contribution of the pennate diatoms in abundance during autumn. The pennate benthic diatoms *Navicula*, *Nitzschia*, *Amphora*, *Pleurosigma*, *Gyrosigma*, *Mastogloia,* and *Diploneis* species were the major forms, while the centric diatoms arranged in chains or aggregated belong to the genera *Chaetoceros* and *Proboscia* were of common occurrence, and the solitary species without cell projections, *Cyclotella* species, were of considerable importance. The shift in relative seasonal contribution among the species in the community varied greatly, which might cause serious ecological impacts on ecosystem function (Magurran, 2004). The species succession indicated repeated dominance of *P*. *alata* f. *gracillima* during winter and spring, and *T. nitzschioides* during summer and autumn. Changes in NH_4_ and SiO_4_ concentrations seem to be pushing this succession (Rousseau et al., 2002; Schlüter et al., 2012). Due to their short life cycle, diatoms respond quickly to a variety of environmental conditions, such as organic pollution (Lobo et al., 2016), and eutrophication (Potapova and Charles, 2007). Since each phytoplankton species has its own characteristic ecophysiological traits that determine how it responds to the environment (Schwaderer et al., 2011), the relative species abundances varied greatly in the same community as commonly expected. Key species, a major constituent of the varied production (Hillebrand et al., 2008), are important sentinels of environmental stress to characterize the different systems (Shi et al., 2012; Jiang et al., 2014). The diatom species *Thalassionema nitzschioides* and *Proboscia alata* f. *gracillima,* species with greater proportional surface area to volume, have the ability for fast uptake of nutrients, likely explaining their dominance (Sigman & Hain, 2012). *Thalassionema nitzschioides,* a pennate, neritic, and cosmopolitan pelagic diatom species, culminated its major peak in the autumn at 20.7-22 °C and 43.9-44.74 psu. Such a temperature range seems preferable for growth in the coastal waters of the Suez Gulf (Nassar, 2000); the author also documented high production in the autumn. Temperature provokes changes in community structure (Nixon et al., 2009), periodicity and abundance of phytoplankton species (Tittensor et al., 2010). Kapkov et al. (2011) reported that *T. nitzschioides* has the ability to transfer to mixotrophic nutrition, allowing for high population growth rates. However, it is hard to recognize the species as a biological indicator of different water quality in the bay because of its wide tolerance to varied environmental conditions (Bonilla et al., 2005). Its blooms were previously reported in indifferent habitats mostly in the summer under a wide range of temperature, salinity, and stratified water column conditions and when SiO_4_ loading was high (e.g. Rajasekar et al., 2010; Pan et al., 2016). The centric neritic diatom *P*. *alata* f. *gracillima* contributed the second-most-significant species, leading the community in the winter and spring. Its major bloom in cold winter seems to share the sharp reduction in nutrient concentrations, falling to their minimal during the whole period, particularly NO_3_ and PO_4_ (1.6-2.63 µg l^-1^ and 1.65-2.5 µg l^-1^). The recurrence of major occurrences of *P. alata* f. *gracillima* might be explained by its ability to benefit from pulsed availability of nutrients, store nutrients, and prosper in environments where nutrients are not available (Phlips et al., 2010). As per the finding, the highly dominated communities by a single and/or couple species reflect disturbances in the ecosystem, such as eutrophication. Moreover, the dinoflagellate *Tripos furca* contributed mainly to the high occurrence and abundance of the genus *Tripos* in the winter and the spring. It is more likely that the dominance of *T. furca* is due to its mixotrophic ability rather than preferential grazing on other *Tripos* species (Tunin-Ley et al., 2007).

Some species: *Pleurosigma formosum, Campylodiscus neofastuosus, Surirella gemma, Leptocylindrus mediterraneus, Tripos californiensis, Tripos furca* var*. brevicornis,* and *Chattonella* cf. *marina,* are not considered Indian Ocean or Red Sea forms. However, these species were recorded from different parts of the Mediterranean Sea and therefore may have transgressed south through the anti-Lessepsian migration (Por, 2012). The community was characterized by the exclusive presence of the Red Sea endemic species *Tripos egyptiacus*, which has not been recorded elsewhere. Chlorophytes, cyanophytes, and euglenophytes had seasonal periods of noticeable occurrence and development that varied within the different ecological factors at the different sites (Marshall et al., 2006). Schabhüttl et al. (2013) reported that green algae showed higher growth at lower temperatures, while cyanophytes show stronger responses with increasing temperatures in mixed communities. The presence of freshwater species, even at low densities; *Oscillatoria, Pediastrum*, *Scenedesmus, Staurastrum*, and *Euglena* species, certainly has a strong connection with freshwater input and organic pollution (Ajayan & Ajit Kumar, 2017). Moreover, some notable succession patterns involved the well-sharing of green and blue-green algae in the spring, when the nitrogen to phosphorus ratio was high (23.41:1.56), in similarity to Barlow et al. (1993). The change in the species composition was also proved by the restricted occurrence of the following species: *Pseudo-nitzschia pungens, Skeletonema costatum, Chaetoceros decipiens*, and *Gyrosigma attenuatum, which* were previously recorded dominants in the bay (Nassar and Hamed, 2003).

**Statistically**, PERMANOVA revealed significant seasonal variability in the phytoplankton community composition (df = 3, MS: 18524, Pseudo-F: 32.88, p-value = 0.001). Also, the value of the R-statistic and significance level of ANOSIM confirmed this significant variation across all seasons (R = 0.995, P = 0.001). Highly significant pair-wise comparisons were detected between seasons. A quasi-symmetric contribution between diatoms and dinoflagellates existed in this variability (ANOSIM R = 0.995, 0.928, P = 0.001, 0.001, respectively).

**Fig. 2:**
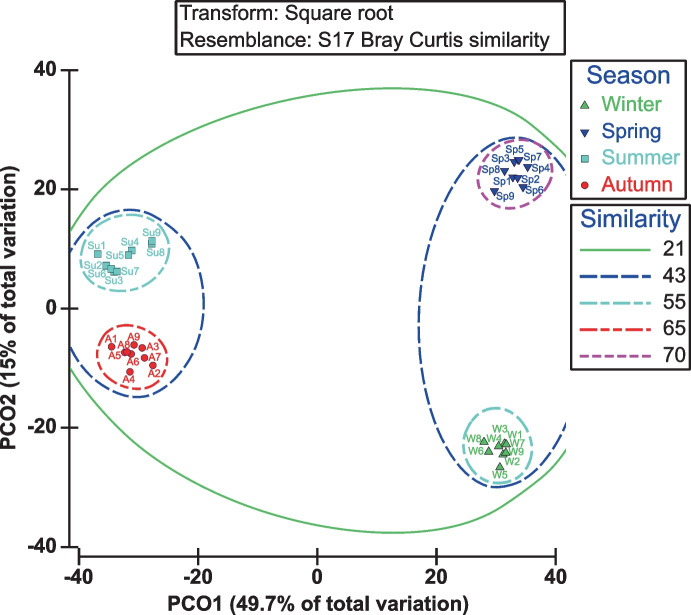
PCO of phytoplankton assemblages in different seasons coupled to a cluster analysis. Solid and dashed lines represent the principal clusters identified as % of Bray Curtis similarity

In addition, the principle coordination analysis (PCO) revealed that the first two axes accounted for 49.7% and 15%, respectively, of the variance in phytoplankton assemblages among seasons. Distinct differentiation was observed between the wet seasons (winter and spring) and dry seasons (summer and autumn) on the multi-dimensional scaling ordination. Both wet and dry seasons contributed almost equally to PCO1 variation, while wet seasons contributed more to PCO2 variation (Fig. 2).

Furthermore, the results of SIMPER analysis show the discrimination between seasons into species contributions. Species are ordered by their average contribution to the average dissimilarity, with highest average dissimilarity between winter and summer (80.52) (Table 2).

**Table 2:** Simper analysis showing the average dissimilarity between different seasons with the main contributing species.

**Winter & Spring Average dissimilarity = 56.56**		**Winter & Summer Average dissimilarity = 80.52**	
**Species **	**Contrib%**	**Species **	**Contrib%**
*Proboscia alata *f. *gracillima*	7.00	*Proboscia alata *f. *gracillim*	11.53
*Prorocentrum gracile*	2.86	*Thalassionema nitzschioide*	8.43
*Scrippsiella trochoidea*	2.23	*Leptocylindrus minimu*	1.76
*Prorocentrum minimum*	1.78	*Phalacroma oxytoxoides*	1.64
*Tryblionella coarctata*	1.65	*Tryblionella coarctata*	1.39
*Chlorella *sp.	1.65	*Amphiprora sulcata*	1.35
*Leptocylindrus minimus*	1.46	*Diplopsalis lenticula*	1.34
*Diplopsalis lenticula*	1.33	*Pseudosolenia calcar-avis*	1.14
**Cumulative contribution %**	19.98	**Cumulative contribution % **	28.59

**Spring & Summer Average dissimilarity = 79.72**		**Winter & Autumn Average dissimilarity = 75.30**	
**Species**	**Contrib%**	**Species**	**Contrib%**
*Thalassionema nitzschioides*	10.18	*Thalassionema nitzschioides*	17.7
*Proboscia alata *f. *gracillima*	5.74	*Proboscia alata *f. *gracillima*	10.43
*Prorocentrum gracile*	4.17	*Thalassionema frauenfeldii*	2.97
*Scrippsiella trochoidea*	3.27	*Phalacroma oxytoxoides*	1.57
*Prorocentrum minimum*	2.52	*Leptocylindrus minimus*	1.19
*Chlorella *sp.	2.16	*Diplopsalis lenticula*	1.11
*Phalacroma oxytoxoide*	1.97	*Pseudosolenia calcar-avi*	1.01
**Cumulative contribution %**	30.01	**Cumulative contribution %**	35.99

**Spring & Autumn Average dissimilarity = 76.82**		**Summer & Autumn Average dissimilarity = 55.12**	
**Species**	**Contrib%**	**Species**	**Contrib%**
*Thalassionema nitzschioides*	19.90	*Thalassionema nitzschioide*	22.82
*Proboscia alata *f. *gracillima*	4.85	*Thalassionema frauenfeldii*	8.41
*Prorocentrum gracile Schütt*	3.64	*Protoperidinium globulus*	1.35
*Thalassionema frauenfeldii*	3.52	*Amphiprora sulcata*	1.31
*Scrippsiella trochoidea*	2.90	*Chaetoceros compressus*	1.18
*Prorocentrum minimum*	1.94	*Chaetoceros pseudocurvisetus*	1.08
**Cumulative contribution %**	36.76	**Cumulative contribution %**	36.15

Moreover, SIMPER analysis showed that the average similarity between winter stations was 66.85%, and only *Proboscia alata* f*. gracillima* contributed 17% of this similarity. Also, the average similarity between spring stations was 72.87%, and *Proboscia alata* f*. gracillima, Prorocentrum gracile,* and *Scrippsiella trochoidea* contributed 19.64% of this similarity, collectively and descending ordered respectively. In addition, the average similarity between summer stations was 57.27%, and *Cylindrotheca closterium* contributed 47.29 and 3.42% of this similarity, respectively. Finally, the average similarity between autumn stations was 72.68%, and *Thalassionema nitzschioides* and *Thalassionema frauenfeldii* contributed 48.46 and 10.71% of this similarity, respectively (Table 3).

**Table 3:** Simper analysis showing the average similarity between different stations in each season with the main contributing species.

**Winter**		**Spring**	
**Average similarity = 66.85**		**Average similarity = 72.87**	
**Species **	**Contrib**%	**Species **	**Contrib**%
*Proboscia alata f. gracillima*	17.08	*Proboscia alata f. gracillima*	8.10
*Phalacroma oxytoxoides*	2.72	*Prorocentrum gracile*	5.90
*Leptocylindrus minimus*	2.36	*Scrippsiella trochoidea*	5.64
*Protoperidinium cerasus*	2.15	*Tripos furca var. eugrammus*	3.45
*Tripos furca var. eugrammus*	2.13	*Protoperidinium cerasus*	2.80
*Tryblionella coarctata*	2.12	*Phalacroma oxytoxoides*	2.80
*Amphiprora sulcata *	2.07	*Prorocentrum minimum*	2.72
**Cumulative contribution %**	30.63	**Cumulative contribution %**	31.4

**Summer**		**Autum**	
**Average similarity = 57.27**		**Average similarity = 72.68**	
**Species**	**Contrib%**	**Species**	**Contrib%%**
*Thalassionema nitzschioides*	47.29	*Thalassionema nitzschioides*	48.46
*Cylindrotheca closterium*	3.42	*Thalassionema frauenfeldii*	10.71
**Cumulative contribution %**	50.71	**Cumulative contribution %**	59.17

### Potentially harmful microalgal species

Among the phytoplankton species identified in this study, several (28 species) potentially harmful phytoplankton species were recorded, of which 19 species of them (67.8% of the total) are dinoflagellates, while diatoms represented by 5 species (See Appendix 1, Table 3). These species were reported elsewhere as bloom-forming or toxin producers or both. For instance, *Tripos furca* and *Tripos fusus* are known as bloom-forming species (Morton et al., 2011; Hallegraeff et al., 2004). The highest cell density of *T. fruca* that was observed in this study was about 1200 cells l^-1^ at st.4 in the spring, which is still a low density for causing hypoxia condition (Yurimoto et al., 2015). *Prorocentrum* and *Phalacroma* are associated with shellfish poisoning (Prabowo & Agusti, 2019). *Gambierdiscus* and *Ostreopsis* are responsible for Ciguatera fish poisoning (CFP); the most commonly reported nonbacterial seafood disease in the world (Catania et al., 2017). Abd-Elhaleem and Abd-Elkarim (2011) reported 280 cases of Ciguatera Fish Poisoning (CFP) with 7 deaths in Egypt in 2007. Even though no red tide blooms have been observed, the presence of such species contributes negatively to ecosystem functioning and represents a serious risk to public health in the future (El-Sheekh et al., 2010; Anderson et al., 2017). Another change in the community detected from the present appearance, even in the low abundance of species that are registered aliens to the bay, might offer extra evidence as effective biological elements in evaluating responses to progressive eutrophication.

### Phytoplankton abundance

The spatial and seasonal variation of phytoplankton abundance progressed in the bay as follows: The phytoplankton density (See Appendix 1, Table 4) varied widely from a minimum of 9530 cells l^-1^ (summer, St. 8) to a maximum of 84265 cells l^-1^ (autumn, St. 2). Station 2 is characterized by arrival and departure of ships, specialized for petroleum oil transportation. This elevated abundance is most likely related to convective overturning of the water column which would provide nutrients to the surface waters (Acker et al., 2008). In addition, based on the mean value, St. 8, the reference station that is quite distant from any pollution source, sustained the lowest mean density (19145 cells l^-1^), and it was highest at St. 7 (40905 cells l^-1^). Station 7 has been influenced by the shipping and trading in the bay. It is a semi-closed area that seems to act as a barrier against the complete water circulation in the bay which allows the accumulation of contaminants. The total phytoplankton density exhibits a high seasonal variation, with autumn the most fertile season (average 70435 cells l^-1^). The maximum abundance was found at the bay areas where the species diversity was low as a result of high nutrient availability, similar to Periyanayagi et al. (2007), a high species number is not so necessarily accompanying high phytoplankton abundance. Compared with the previous data in Gulf of Suez (Madkour et al., 2010), and in the Suez Bay (Nassar et al., 2016), the present maximum density is greater by 1.62-8.52 fold increase, representing an indicator for progressive eutrophication development. Based on the scale of Kitsiou and Karydis (2001, 2002) of phytoplankton abundance for water status, the water in the bay could be described a mesotrophic system.

### Chlorophyll *a*

**Fig. 3:**
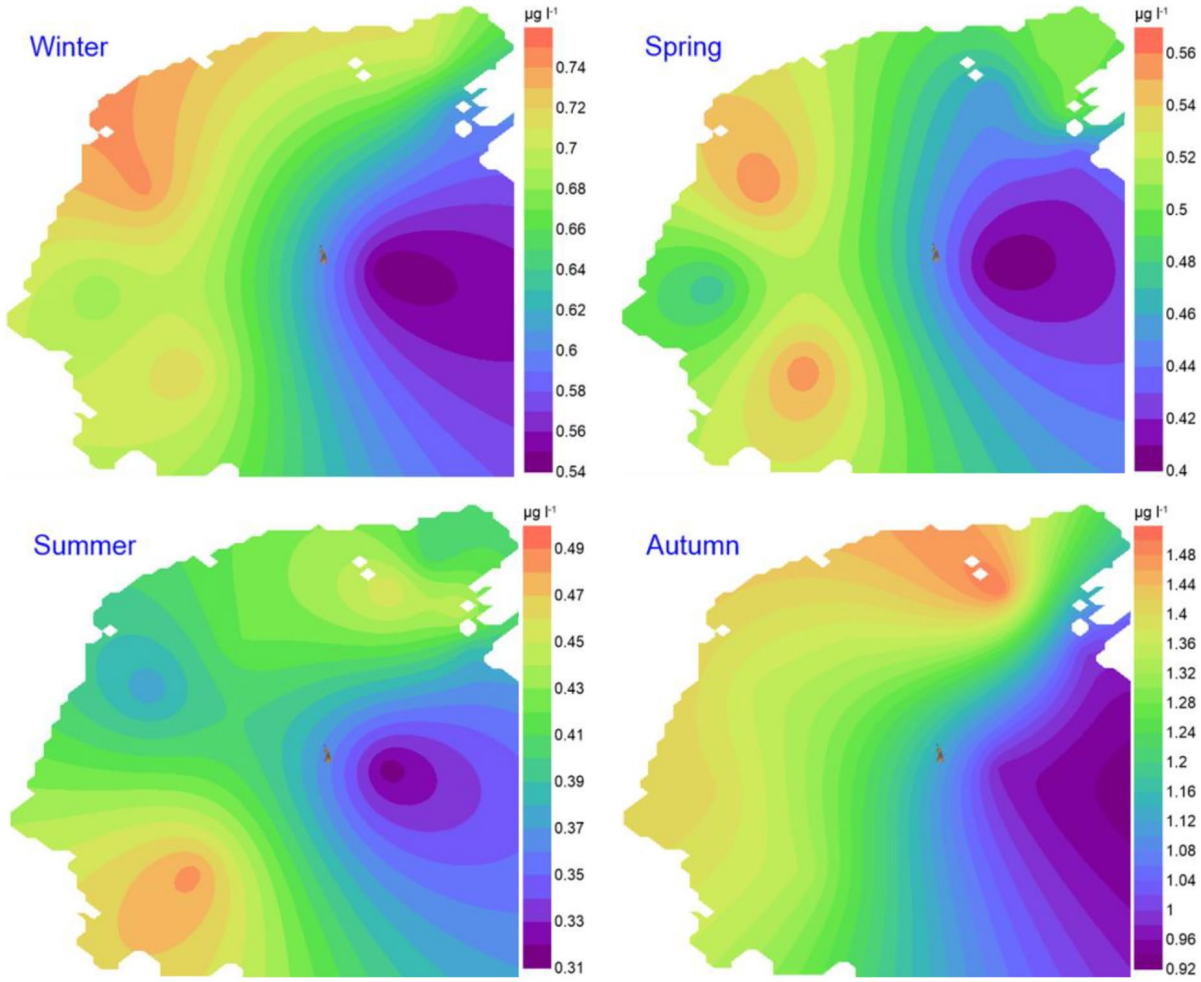
Spatiotemporal distributions of chlorophyll *a* concentration (µg. l^-1^)

 The seasonal concentration of chlorophyll *a* concentrations did not exceed 1.5 µg l^-1^; the highest in the autumn and the lowest at the reference station in the summer, and there was a limited difference between stations during the same sampling day. The magnitude of Chl. *a* concentration can be arranged generally in the order as autumn > winter > spring > summer(See Appendix 1, Table 4). The spatial distribution reflects similar patterns in the winter and autumn when the rich layer occupied the near-shore areas towards the west, and it was expanding wider in the last season. The patterns were different in the spring and summer, explaining the inner bay water of relatively lower Chl. *a* that occupy most of the bay, and extends further to reach the inshore areas in the latter season (Fig. 3).

### Phytoplankton diversity

**Fig. 4:**
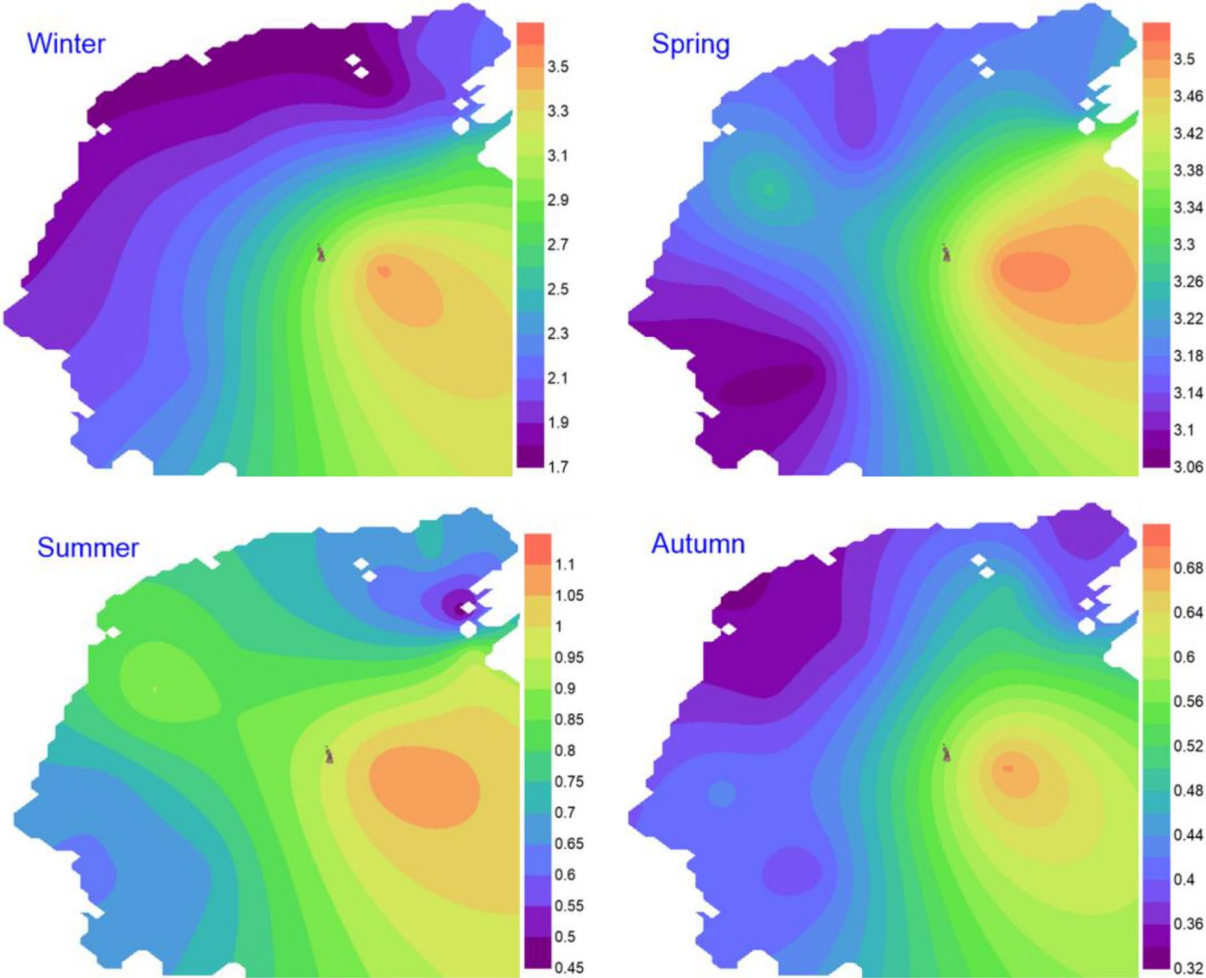
Spatiotemporal distributions of Shannon diversity index in Suez Bay

The values of Pielou's evenness (*J*') fluctuated between a minimum of 0.08 (St.5, autumn) and a maximum of 0.82 (St.8, spring). Similarly, the minimum value of Shannon diversity (0.35) was observed at St.5, autumn, and the maximum of 3.52 at St.8, winter (See Appendix 1, Table 5). The spatial distribution patterns of the Shannon diversity values during the four seasons show that the entire bay seems to exhibit much higher values (Fig. 4). On the other hand, to describe temporal beta diversity, a measure of species turnover across time in Suez Bay was done. Based on the values of modified Whittaker (β_W_) and beta turnover (β_T_) (Table 4) that measure the inverse of the average frequency of species, the results indicate that the species turnover among the four seasons could be arranged in order of magnitude as winter > summer > autumn > spring.

**Table 4:** Temporal β diversity of the phytoplankton assemblages during the four seasons in Suez Bay

	**Winter**	**Spring**	**Summer**	**Autumn**
Species richness α	278	152	196	179
Gained species g(H)	107	25	52	35
Common species	171	127	144	144
Lost species l(H)	145	271	227	244
Whittaker's species turnover (βW)	0.522	1.783	1.158	1.363
Beta turnover (βT)	0.453	0.974	0.712	0.779

### Environmental factors controlling phytoplankton community structure

The factors shaping the phytoplankton dynamics and variability in the bay were numerous, each influencing the other. Such influence has a major impact on temporal turnover in phytoplankton composition, or succession (Calić et al., 2013). Phytoplankton abundance (*N*) and Chl. *a* concentration, was significantly positively correlated with pH, salinity, PO_4_, NO_3_, DO and BOD, while phytoplankton diversity, in terms of species richness (S), Pielou's evenness and Shannon indexes, was negatively correlated with these parameters. The significant negative correlations of NO_3_ and PO_4_ with evenness go in parallel with the results of Gamfeldt and Hillebrand (2011) and Hillebrand and Lehmpfuhl (2011). In addition, the temperature was negatively correlated with both species richness and abundance, while COD was negatively correlated with phytoplankton diversity and positively correlated with abundance (See Appendix 1, Table 6). Moreover, SiO_4_ was negatively correlated with both diatoms abundance and species richness (Pearson R = -0.33, -0.39, P-value = 0.04, 0.01, respectively).

It is known that temperature variations (Tittensor et al., 2010), and salinity (Flöder & Burns, 2004) contribute effectively to the different components of the phytoplankton community, and the nutrients; phosphate (Mackey et al., 2007), nitrate (Howarth & Marino, 2006), and silicate as well (Olli et al., 2008). The multiple regression analysis showed that pH, water temperature, salinity, NO_3_ and NO_2_ were the most important explanatory parameters in regard to phytoplankton abundance (*N*) and Chl. *a* explaining 83.6 and 88.5% of their variabilities, respectively. The relation is expressed as:

$$\begin{aligned}N\;&=\;-400679+35921\;\ast PH-4319\;\ast Temp.+5431\\&\;\ast Sal.+1670\;\ast NO_3\;(R^2=0.836,\;p<0.0001)\end{aligned}$$

$$\begin{aligned}Chl.\;a\;&=\;-9.21+0.98\;\ast PH-0.0768\;\ast Temp.+0.0766\\&\;\ast Sal.+0.182\;\ast NO_2\;(R^2=0.885,\;p<0.0001)\end{aligned}$$

**Fig. 5:**
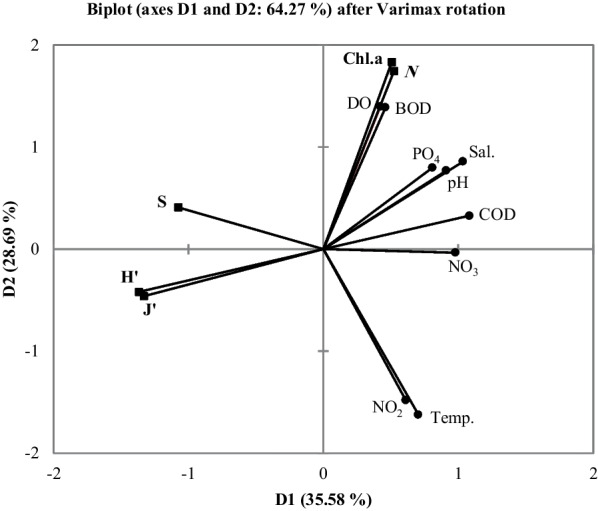
Principal component analyses (PCA) biplot for the relationship between environmental factors and Shannon (H'),Pielou's evenness (J'), species number (S), total individuals (*N*), and chlorophyll *a*

The principal component analyses (PCA) stressed the importance of the previously mentioned parameters in governing phytoplankton biomass and diversity (Fig. 5).

### Stability of phytoplankton community

The temporal stability indexes (TSI) fluctuated within a narrow range, > 2 at Sts. 1, 7, and 9, and almost similar values at the other stations fluctuating between 1.56 at St. 2 and 1.9 at St. 8; the higher value indicates higher stability (Table 5).

**Table 5:** Calculated phytoplankton stability index (TSI), mean values and standard deviation (SD) of chlorophyll *a* concentration (µg l^-1^)

**Station**	**St. 1**	**St. 2**	**St. 3**	**St. 4**	**St. 5**	**St. 6**	**St. 7**	**St. 8**	**St. 9**
**Mean (Chl. *a***)	0.67	0.77	0.73	0.75	0.76	0.75	0.78	0.56	0.6
**SD (Chl. *a***)	0.31	0.5	0.42	0.42	0.42	0.45	0.39	0.29	0.28
**TSI**	2.15	1.56	1.76	1.77	1.78	1.65	2.01	1.9	2.14

The results showed that DO, NH_4_ and NO_2_ were significantly correlated with stability and chlorophyll *a*, while BOD, which strongly affected stability, shows an insignificant correlation with chlorophyll *a* (negative correlation). In addition, the results concerning the SD of environmental factors as expressing variability go in parallel with the mean values, explaining the importance of nitrogen elements, and salinity seems to be the main contributory stressing the temporal stability destabilization. Variations in phosphate and silicate contributed significantly to TSI and Chl. *a*, respectively (See Appendix 1, Table **7**). Our results were consistent with findings of Tian et al. (2016).

**Fig. 6:**
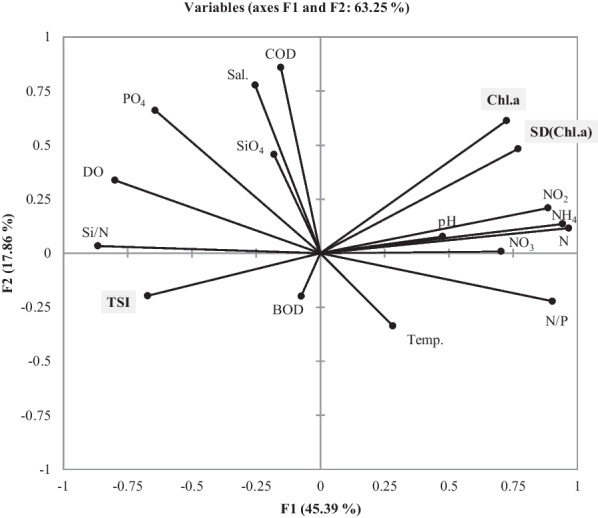
Principal components analysis (PCA) biplot for the relationships between environmental factors and phytoplankton stability index (TSI)

The PCA for the environmental factors revealed that the first and second principal components explained 45.39% (eigenvalues = 7.7) and 17.86% (eigenvalues = 3), respectively, of the variance for all the variables (Fig. 6). Phytoplankton TSI was significantly positively correlated with both the mean values of DO and Si/N ratio and the variability (SD) of salinity, NO_2_, and PO_4_**.** However, phytoplankton TSI was significantly negatively correlated with the mean values of NH_4_, NO_2_, and thereby N/P ratio.

## Eutrophication assessment

The TRIX index can be useful in quantifying environmental quality and the eutrophication state with values varying from 0 to 10, ranging from oligotrophic to eutrophic conditions (Cloern, 2001). The trophic index (TRIX) values ranged between 3.2 (spring, St. 8) and 4.7 (autumn, St. 2). The seasonal variation fluctuated between 3.55 ± 0.07 and 4.1 ± 0.11 in the spring and autumn, respectively. Regionally, the lowest mean trophic index value (3.48 ± 0.108) was observed at St. 8, while the highest (3.904 ±0.168) was observed at St. 7 during the four seasons (Table 6). The TRIX values indicate dominance of mesotrophic (moderate water quality) status, particularly in the winter, spring, and summer except for St. 6 and 7 in the summer that exhibited poor water quality (eutrophic); the stations have been affected by ship-based activities, industrial drain and sewage drains from the small communities. The eutrophic condition occupied the almost investigated area in the autumn, except at St. 8 (reference), St. 5, and 6. The higher values observed for the TRIX index in the autumn were due to the increased phytoplankton abundance, and thereby chlorophyll *a* concentration, besides other eutrophication parameters (total nitrogen and phosphorus).

**Table 6:**
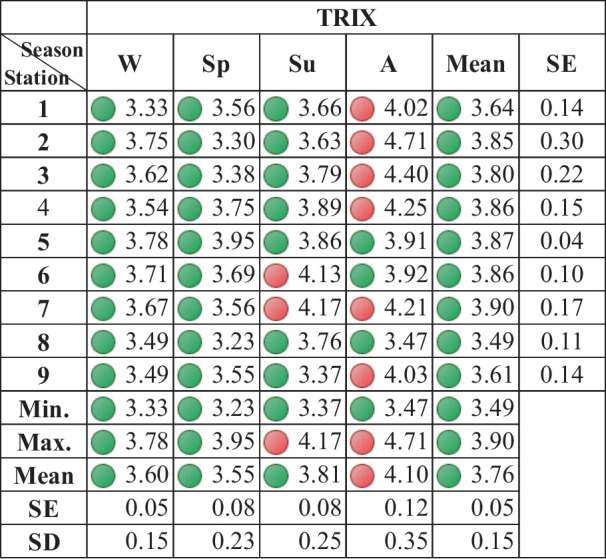
The trophic index (TRIX) values for the sampling stations. (● Red icon: eutrophic, ● Green icon: mesotrophic level)

## Conclusion

The phytoplankton community structure in Suez Bay is highly diverse. It varied significantly among different seasons, and diatoms mainly contribute almost equally with dinoflagellates to this variability. The species richness comprised 423 species belonging to 11 groups and was much more than the previous records in the bay. The species turnover among the four seasons could be arranged in order of magnitude as winter > summer > autumn > spring, while the magnitude of Chl. *a* concentration and phytoplankton abundance, can be arranged generally in the order of autumn > winter > spring > summer. The high degree of diatom species dominance was of paramount importance to the bay community, followed by dinoflagellates; diatoms dominated most of the time, while dinoflagellates were more abundant in the spring. The study expresses the power of multifactor influences on community structure to agree with the so-called co-limitation paradigm, where the influence of nutrients is addressed in combination. The mean values of DO and variability of salinity had a positive influence on the phytoplankton stability, while the effects of major nitrogen sources were always negative. The existence of several potentially harmful species has a negative impact on water quality and is considered "red flags" for such ecosystems threatened by a variety of anthropogenic impacts. The bay water was classified as mesotrophic, moderately polluted, almost the entire year, except at the end of the year, when it became eutrophic**.** This indicates "semi-sensitive" ecosystems that can be ‘sensitive’ and then "eutrophic" in the future with the potential increase of human impacts.

### Supplementary Information

Below is the link to the electronic supplementary material.

Supplementary file1 (DOCX 380 KB)

## Data Availability

The datasets used can be accessed can be available upon request from the second author.
