# Supplementary material for: Seasonal dynamics of phytoplankton in the northern part of Suez Gulf, Egypt
Source: Environ Monit Assess. 2023 Aug 17;195(9):1060. doi: 10.1007/s10661-023-11688-7 (PMC10435403; doi:10.1007/s10661-023-11688-7)
Supplement: Supplementary file 1 — Supplementary file1 (DOCX 380 KB) [file 10661_2023_11688_MOESM1_ESM.docx]

Table 1: Seasonal variations of the measured physicochemical parameters of the Suez Bay surface water. A: autumn, Su: summer, Sp: spring, W: winter, SE: standard error, SD: standard deviation

**Table 2:** Molar ratio of total nitrogen to phosphate concentrations and silicate to total nitrogen concentrations in Suez Bay.

* limited by N. ** co-limited by N and P. *** limited by P. **^+^** limited by Si.

**Table 3:** List and seasonal frequency distribution of the recorded phytoplankton species in Suez Bay.

| **Sampling station   Phytoplankton species** | **Winter** | **Spring** | **Summer** | **Autumn** |
| --- | --- | --- | --- | --- |
| **Diatoms** |  |  |  |  |
| **Centric diatoms** |  |  |  |  |
| *Actinocyclus octonarius* Ehrenberg | ─ | X | X | X |
| *Arachnodiscus ehrenbergii* J.W. Bailey * | X | ─ | ─ | ─ |
| *Asteromphalus flabellatus* (Brébisson) Grevill | ─ | ─ | X | ─ |
| *Aulacoseira granulata* (Ehrenberg) Simonsen | X | X | X | ─ |
| *Aulacoseira italica* (Ehrenberg) Simonsen | X | ─ | ─ | ─ |
| *Biddulphia alternans* (Bailey) Van Heurck * | ─ | ─ | X | ─ |
| *Biddulphia tridens* (Ehrenberg) Ehrenberg | X | X | X | X |
| *Campylodiscus* cf. *clypeus* (Ehrenberg) Ehrenberg ex Kützing * | X | ─ | ─ | ─ |
| *Campylodiscus echeneis* Ehrenberg ex Kützing * | X | ─ | ─ | ─ |
| *Campylodiscus noricus* Ehrenberg ex Kützing | X | X | X | X |
| *Cerataulina bicornis* (Ehrenberg) Hasle | X | ─ | ─ | ─ |
| *Cerataulina pelagica* (Cleve) Hendey ● | ─ | X | ─ | X |
| *Cerataulus radiatus* R.Ross * | ─ | ─ | X | ─ |
| *Chaetoceros affinis* Lauder | X | X | X | X |
| *Chaetoceros anastomosans* Grunow | ─ | ─ | ─ | X |
| *Chaetoceros atlanticus* Cleve | X | ─ | ─ | ─ |
| *Chaetoceros borealis* Bailey | ─ | ─ | X | ─ |
| *Chaetoceros coarctatus* Lauder | X | X | X | X |
| *Chaetoceros compressus* Lauder | ─ | ─ | ─ | X |
| *Chaetoceros decipiens* Cleve | X | X | X | ─ |
| *Chaetoceros densus* (Cleve) Cleve | X | ─ | ─ | ─ |
| *Chaetoceros difficilis* Cleve * | X | ─ | ─ | ─ |
| *Chaetoceros laciniosus* F.Schütt | ─ | ─ | ─ | X |
| *Chaetoceros lorenzianus* Grunow | X | ─ | ─ | ─ |
| *Chaetoceros peruvianus* Brightwell | X | ─ | X | ─ |
| *Chaetoceros pseudocurvisetus* Mangin | X | X | X | X |
| *Chaetoceros simplex* Ostenfeld | X | ─ | ─ | ─ |
| *Chaetoceros teres* Cleve * | X | ─ | ─ | ─ |
| *Chaetoceros tortissimus* Gran | ─ | ─ | X | X |
| *Corethron criophilum* Castracane | ─ | ─ | X | ─ |
| *Coscinodiscus centralis* Ehrenberg | ─ | X | X | ─ |
| *Coscinodiscus granii* L.F.Gough | X | X | X | ─ |
| *Coscinodiscus wailesii* Gran & Angst * | X | ─ | ─ | ─ |
| *Cyclophora* cf*. castracanei* Ashworth & Lobban * | ─ | ─ | X | ─ |
| *Cyclotella meneghiniana* Kützing | ─ | ─ | X | X |
| *Cyclotella polymorpha* B.Meyer & Håkansson * | X | ─ | X | X |
| *Cyclotella* sp. | X | X | X | ─ |
| *Cyclotella striata* (Kützing) Grunow * | X | ─ | X | X |
| *Cyclotella stylorum* Brightwell * | ─ | ─ | X | X |
| *Eucampia zodiacus* Ehrenberg * | ─ | ─ | X | ─ |
| *Guinardia delicatula* (Cleve) Hasle in Hasle & Syvertsen | X | ─ | ─ | X |
| *Guinardia flaccida* (Castracane) H.Peragallo | X | X | X | X |
| *Guinardia striata* (Stolterfoth) Hasle | X | X | ─ | X |
| *Hemiaulus hauckii* Grunow ex Van Heurck | X | X | ─ | ─ |
| *Hemiaulus membranaceus* Cleve | X | X | ─ | ─ |
| *Hemiaulus sinensis* Greville | X | X | ─ | X |
| *Lampriscus orbiculatus* (Shadbolt) Peragallo & Peragallo * | X | ─ | ─ | ─ |
| *Lauderia annulata* Cleve | X | ─ | ─ | X |
| *Leptocylindrus danicus* Cleve | X | ─ | X | X |
| *Leptocylindrus mediterraneus* (Peragallo) Hasle | ─ | ─ | X | X |
| *Leptocylindrus minimus* Gran | X | X | X | X |
| *Lithodesmioides polymorpha* var*. polymorpha* Stosch * | X | ─ | ─ | ─ |
| *Lithodesmioides polymorpha* var. Stosch * | X | ─ | ─ | ─ |
| *Lithodesmioides polymorphum* Stosch | X | ─ | ─ | ─ |
| *Neocalyptrella robusta* (G.Norman ex Ralfs) Hernández-Becerril & Meave del Castillo | X | ─ | ─ | ─ |
| *Odontella aurita* (Lyngbye) C.Agardh | X | ─ | ─ | ─ |
| *Odontella obtusa* Kützing | ─ | ─ | X | X |
| *Palmerina hardmaniana* G.R.Hasle * | ─ | X | ─ | ─ |
| *Paralia sulcata* (Ehrenberg) Cleve | X | X | X | X |
| *Perissonoë cruciata* (Janisch & Rabenhorst) Andrews & Stoelzel * | ─ | ─ | X | ─ |
| *Podosira stelligera* (Bailey) A.Mann * | X | X | ─ | ─ |
| *Porosira glacialis* (Grunow) Jørgensen * | ─ | ─ | X | ─ |
| *Proboscia alata* (Brightwell) Sundström | X | X | ─ | ─ |
| *Proboscia alata* f. *gracillima*(Cleve) Gran | A | R | X | X |
| *Proboscia indica* (H.Peragallo) Hernández-Becerril | X | X | ─ | X |
| *Pseudosolenia calcar-avis* (Schultze) B.G.Sundström | X | X | ─ | X |
| *Rhizosolenia imbricata* Brightwell | X | ─ | X | X |
| *Rhizosolenia imbricata* var. *shrubsolei*(Cleve) Schröder | X | X | ─ | X |
| *Rhizosolenia styliformis* T.Brightwell | X | ─ | X | X |
| *Roperia tesselata* (Roper) Grunow ex Pelletan | X | ─ | ─ | ─ |
| *Skeletonema costatum* (Greville) Cleve | ─ | ─ | X | ─ |
| *Thalassiosira angustelineata* (A.W.F.Schmidt) G.Fryxell & Hasle | ─ | ─ | X | X |
| *Thalassiosira eccentrica* (Ehrenberg) Cleve | X | ─ | X | X |
| *Thalassiosira oestrupii* (Ostenfeld) Proschkina-Lavrenko ex Hasle | ─ | ─ | X | X |
| *Thalassiosira punctigera* (Castracane) Hasle * | ─ | ─ | X | X |
| *Thalassiosira* sp. | ─ | X | X | ─ |
| *Thalassiosira subtilis* (Ostenfeld) Gran | ─ | ─ | X | X |
| *Triceratium robertsianum* Greville * | X | ─ | ─ | ─ |
| *Trieres mobiliensis* (Bailey) Ashworth & Theriot | X | ─ | X | X |
| *Trieres regia* (M.Schultze) M.P.Ashworth & E.C.Theriot * | ─ | ─ | X | X |
| *Trieres sinensis* (Greville) M.P.Ashworth & E.C.Theriot | ─ | ─ | X | ─ |
| **Pennate diatoms** |  |  |  |  |
| *Achnanthes brevipes* C.Agardh * | X | ─ | ─ | ─ |
| *Amphora arenaria* Donkin * | X | ─ | ─ | ─ |
| *Amphora egregia* Ehrenberg * | X | ─ | ─ | ─ |
| *Amphora lineolata* Ehrenberg | ─ | X | X | X |
| *Amphora marina* W.Smith | ─ | X | X | ─ |
| *Amphora ocellata* Donkin * | X | ─ | ─ | X |
| *Amphora ovalis* (Kützing) Kützing | X | ─ | ─ | X |
| *Amphora proteus* W.Gregory * | X | X | ─ | X |
| *Amphora quadrata* Brébisson ex Kützing * | X | ─ | ─ | ─ |
| *Amphora* sp.1 | ─ | ─ | X | X |
| *Amphora* sp.2 | ─ | ─ | X | ─ |
| *Amphora spectabilis* W.Gregory * | X | X | X | ─ |
| *Asterionella formosa* Hassall * | X | ─ | ─ | ─ |
| *Asterionellopsis glacialis* (Castracane) Round | X | ─ | X | X |
| *Bacillaria paxillifera* (O.F.Müller) T.Marsson | X | X | ─ | X |
| *Caloneis liber* (W.Smith) Cleve * | X | X | ─ | X |
| *Campylodiscus neofastuosus* Ruck & Nakov | X | X | X | X |
| *Climacosphenia moniligera* Ehrenberg | ─ | ─ | X | ─ |
| *Cocconeis placentula* Ehrenberg | X | X | X | X |
| *Cochlearisigma maroccanum* (Cleve ex H.Paragallo) G.Reid * | ─ | X | ─ | ─ |
| *Craticula cuspidata* (Kutzing) D.G.Mann * | X | ─ | ─ | X |
| *Cylindrotheca closterium* (Ehrenberg) Reimann & J.C.Lewin | X | X | X | X |
| *Cymatopleura solea* (Brébisson) W.Smith * | X | X | X | X |
| *Diploneis bombus* (Ehrenberg) Ehrenberg * | X | X | X | X |
| *Diploneis chersonensis* (Grunow) Cleve * | X | ─ | ─ | ─ |
| *Diploneis crabro* var. *pandura*(Brébisson) Cleve | ─ | ─ | ─ | X |
| *Diploneis didyma* (Ehrenberg) Ehrenberg | X | X | ─ | X |
| *Diploneis fusca* var. *aestiva*(Donkin) Hustedt * | X | X | ─ | ─ |
| *Diploneis incurvata* (Gregory) Cleve * | ─ | X | ─ | ─ |
| *Diploneis interrupta* (Kützing) Cleve | X | X | X | X |
| *Diploneis littoralis* (Donkin) Cleve * | X | X | ─ | X |
| *Diploneis smithii* (Brébisson) Cleve * | X | X | ─ | ─ |
| *Entomoneis alata* (Ehrenberg) Ehrenberg | X | ─ | X | X |
| *Entomoneis gigantea*var*. sulcata*(O'Meara) Moreno, Licea & Santoyo, nom. inval. * | X | X | X | X |
| *Entomoneis paludosa* var. *duplex* (Donkin) Makarova & Achmetova * | X | ─ | ─ | ─ |
| *Entomoneis pseudoduplex* K.Osada & H.Kobayasi * | ─ | ─ | X | ─ |
| *Fallacia* sp. | ─ | ─ | X | ─ |
| *Fragilariopsis* cf*. oceanica* (Cleve) Hasle * | X | ─ | X | ─ |
| *Gyrosigma acuminatum* (Kützing) Rabenhorst | X | X | X | X |
| *Gyrosigma attenuatum* (Kützing) Rabenhorst | X | X | X | X |
| *Gyrosigma balticum* (Ehrenberg) Rabenhorst | X | X | X | X |
| *Gyrosigma delicatulum* (W.Smith) Griffith & Henfrey * | X | ─ | ─ | ─ |
| *Gyrosigma diminutum* (W.Smith) Cleve * | ─ | ─ | X | X |
| *Gyrosigma lineare* (Grunow) Cleve * | ─ | X | ─ | ─ |
| *Gyrosigma obscurum* (W.Smith) J.W.Griffith & Henfrey * | X | ─ | ─ | ─ |
| *Gyrosigma spenceri* var. *arnotti* (Cleve & Grunow) Schulz * | X | ─ | ─ | ─ |
| *Gyrosigma spenceri* (Bailey ex Quekett) Griffith & Henfrey * | X | X | ─ | ─ |
| *Halamphora coffeiformis* (C.Agardh) Mereschkowsky ● | X | ─ | ─ | ─ |
| *Halamphora* cf. *exigua* (W.Gregory) Levkov * | X | ─ | X | ─ |
| *Halamphora hyalina* (Kützing) Rimet & R.Jahn | ─ | ─ | X | ─ |
| *Haslea gigantea* var. *tenuis*von Stosch * | ─ | ─ | X | X |
| *Iconella capronii* (Brébisson & Kitton) Ruck & Nakov | X | ─ | X | ─ |
| *Licmophora abbreviata* C.Agardh | X | ─ | X | X |
| *Licmophora flabellata* (Greville) C.Agardh | X | ─ | ─ | X |
| *Licmophora grandis* (Kützing) Grunow * | ─ | ─ | X | ─ |
| *Licmophora remulus* (Grunow) Grunow * | X | ─ | ─ | ─ |
| *Lyrella fogedii* Witkowski, Lange-Bertalot & Metzeltin * | X | ─ | ─ | ─ |
| *Lyrella hennedyi* (W.Smith) Stickle & D.G.Mann * | ─ | ─ | X | X |
| *Lyrella lyra* var. *recta* f. *subelliptica* (Greville) Peragallo & Peragallo * | X | ─ | ─ | ─ |
| *Lyrella lyra* (Ehrenberg) Karajeva | X | X | X | ─ |
| *Lyrella lyra* var. *subcarinata*(Grunow) Moreno * | X | ─ | ─ | X |
| *Lyrella lyroides* (Hendey) D.G.Mann | ─ | ─ | ─ | X |
| *Mastogloia affirmata* (Leudiger-Fortmorel) Cleve * | X | ─ | ─ | ─ |
| *Mastogloia apiculata* W.Smith * | ─ | ─ | X | ─ |
| *Mastogloia braunii* f. *major* Peragallo & Peragallo * | X | ─ | ─ | ─ |
| *Mastogloia erythraea* Grunow | ─ | ─ | X | ─ |
| *Mastogloia graciloides* Hustedt * | X | ─ | ─ | ─ |
| *Mastogloia lanceolata* Thwaites ex W.Smith * | ─ | ─ | X | ─ |
| *Mastogloia peragalli* Cleve * | X | ─ | ─ | ─ |
| *Mastogloia quinquecostata* Grunow * | X | ─ | ─ | ─ |
| *Mastogloia quinquecostata* var. *elongata* (Leuduger-Fortmorel) Cleve * | X | ─ | ─ | ─ |
| *Navicula bottnica* Grunow in Cleve & Grunow * | ─ | X | ─ | ─ |
| *Navicula cincta* (Ehrenberg) Ralfs * | X | X | X | X |
| *Navicula cryptocephala* Kützing | ─ | ─ | X | ─ |
| *Navicula digitoradiata* (W.Gregory) Ralfs * | ─ | X | X | X |
| *Navicula directa* (Smith) Ralfs | ─ | ─ | X | ─ |
| *Navicula distans* (W.Smith) Ralfs | X | X | X | X |
| *Navicula gregaria* Donkin * | X | ─ | ─ | X |
| *Navicula incerta* Grunow * | X | ─ | ─ | X |
| *Navicula libonensis* Schoeman * | X | ─ | X | ─ |
| *Navicula ramosissima* (C.Agardh) Cleve * | X | ─ | ─ | X |
| *Navicula* sp. | X | X | ─ | ─ |
| *Navicula transitans* Cleve * | X | ─ | ─ | X |
| *Navicula transitans* f. *delicatula*Heimdal * | X | ─ | ─ | ─ |
| *Navicula transitans* var. *derasa*(Grunow) Cleve * | ─ | ─ | X | ─ |
| *Navicula tripunctata* (O.F.Müller) Bory * | X | ─ | ─ | ─ |
| *Nitzschia acicularis* (Kützing) W.Smith | X | ─ | ─ | ─ |
| *Nitzschia* cf*. stellata* Manguin * | ─ | ─ | ─ | X |
| *Nitzschia longissima* (Brébisson) Ralfs | X | ─ | X | ─ |
| *Nitzschia lorenziana* Grunow | X | X | ─ | X |
| *Nitzschia lorenziana* var. *subtilis*Grunow * | X | ─ | ─ | ─ |
| *Nitzschia palea* (Kützing) W.Smith | X | ─ | ─ | X |
| *Nitzschia recta* Hantzsch ex Rabenhorst * | X | ─ | ─ | ─ |
| *Nitzschia reversa* W.Smith * | X | ─ | ─ | X |
| *Nitzschia rigida* var. *rigidula* Peragallo & Peragallo * | X | ─ | ─ | ─ |
| *Nitzschia sigma* (Kützing) W.Smith | X | X | X | X |
| *Nitzschia sigma* var. *intercedens*Grunow * | X | ─ | ─ | ─ |
| *Nitzschia sigma* var. *sigmatella*Grunow * | ─ | ─ | ─ | X |
| *Nitzschia sigmoidea* (Nitzsch) W.Smith * | X | ─ | ─ | X |
| *Nitzschia* sp. | X | ─ | ─ | ─ |
| *Nitzschia spathulata* Brébisson ex W.Smith * | X | X | ─ | X |
| *Petrodictyon gemma* (Ehrenberg) D.G.Mann | ─ | ─ | ─ | X |
| *Petroneis granulata* D.G.Mann * | X | ─ | ─ | X |
| *Petroneis marina* (Ralfs) D.G.Mann | ─ | X | X | X |
| *Pinnularia rectangulata* (W.Gregory) Rabenhorst * | ─ | ─ | X | ─ |
| *Plagiotropis lepidoptera* (W.Gregory) Kuntze | X | ─ | X | ─ |
| *Pleurosigma acutum* Norman ex Ralfs * | ─ | X | ─ | ─ |
| *Pleurosigma angulatum* (J.T.Quekett) W.Smith | X | X | X | X |
| *Pleurosigma directum* Grunow | X | ─ | ─ | ─ |
| *Pleurosigma diversestriatum* F.Meister | X | X | X | X |
| *Pleurosigma elongatum* W.Smith | X | ─ | X | X |
| *Pleurosigma formosum* W.Smith | X | X | X | ─ |
| *Pleurosigma lanceolatum* Donkin * | X | X | X | X |
| *Pleurosigma naviculaceum* Brébisson * | X | X | ─ | ─ |
| *Pleurosigma normanii* Ralfs | X | X | ─ | X |
| *Pleurosigma salinarum* (Grunow) Grunow | X | ─ | ─ | X |
| *Pleurosigma strigosum* W.Smith | X | X | X | X |
| *Psammodictyon panduriforme* (W.Gregory) D.G.Mann | X | ─ | X | X |
| *Psammodictyon panduriforme* var. *minor*(Grunow) E.Y.Haworth & M.G.Kelly | X | X | X | X |
| *Pseudo-nitzschia delicatissima* (Cleve) Heiden ● | X | ─ | ─ | ─ |
| *Pseudo-nitzschia pungens* (Grunow ex Cleve) Hasle ● | X | ─ | ─ | ─ |
| *Pseudo-nitzschia seriata* (Cleve) H.Peragallo ● | X | X | ─ | ─ |
| *Pseudo-nitzschia* sp. | ─ | ─ | X | ─ |
| *Rhoicosigma oceanicum* H.Peragallo * | X | ─ | ─ | ─ |
| *Stenopterobia sigmatella* (W.Gregory) R.Ross * | ─ | ─ | ─ | X |
| *Striatella unipunctata* (Lyngbye) C.Agardh | X | ─ | ─ | ─ |
| *Surirella* cf. *fluminensis* Grunow * | ─ | ─ | X | ─ |
| *Surirella* cf. *hybrida* Grunow * | X | ─ | ─ | ─ |
| *Surirella ovata* Kützing | X | X | X | X |
| *Synedrosphenia fulgens* (Greville) Lobban & Ashworth * | ─ | ─ | X | ─ |
| *Thalassionema frauenfeldii* (Grunow) Tempère & Peragallo | X | X | X | X |
| *Thalassionema nitzschioides* (Grunow) Mereschkowsky | X | X | V | V |
| *Thalassiophysa hyalina* (Greville) Paddock & P.A.Sims * | X | ─ | ─ | ─ |
| *Thalassiophysa rhipidis* Conger * | X | ─ | ─ | ─ |
| *Trachyneis antillarum* (Cleve & Grunow) Cleve * | X | X | ─ | X |
| *Trachyneis aspera* (Ehrenberg) Cleve | X | X | X | X |
| *Trachyneis aspera var. robusta* (Petit) Cleve * | ─ | ─ | X | X |
| *Trachyneis debyi* (Leuduger-Fortmorel) Cleve * | ─ | ─ | ─ | X |
| *Trachyneis oblonga* (Bailey) H.Peragallo & M.Peragallo * | ─ | ─ | ─ | X |
| *Tryblionella coarctata* (Grunow) D.G.Mann * | X | ─ | X | X |
| *Tryblionella littoralis* (Grunow) D.G.Mann | X | ─ | ─ | ─ |
| *Tryblionella marginulata* (Grunow) D.G.Mann * | ─ | ─ | ─ | X |
| *Ulnaria ulna* (Nitzsch) Compère | X | X | X | X |
| **Dinoflagellates** |  |  |  |  |
| *Alexandrium minutum* Halim * ● | ─ | ─ | ─ | X |
| *Amphidinium crassum* Lohmann * | ─ | X | ─ | ─ |
| *Amyloodinium amylaceum* (Bargoni) Brown & Hovasse * | X | ─ | ─ | ─ |
| *Ceratocorys bipes* (Cleve) Kofoid | X | ─ | ─ | ─ |
| *Ceratocorys gourretii* Paulsen | X | ─ | ─ | ─ |
| *Ceratocorys horrida* Stein | X | ─ | ─ | ─ |
| *Chytriodinium* sp. Chatton * | X | ─ | ─ | ─ |
| *Cochlodinium* sp. * ● | ─ | X | X | ─ |
| *Corythodinium diploconus* (Stein) F.J.R.Taylor * | ─ | ─ | X | ─ |
| *Dinophysis acuminata* Claparède & Lachmann ● | X | ─ | X | X |
| *Dinophysis caudata* Saville-Kent var. *pedunculata* (Schmidt) Schröd ● | X | X | X | X |
| *Dinophysis rapa* (F.Stein) Abé | ─ | X | ─ | ─ |
| *Diplopsalis lenticula* Bergh | X | X | X | X |
| *Gambierdiscus toxicus* Adachi & Fukuyo * ● | X | ─ | X | ─ |
| *Gyrodinium* sp. | ─ | ─ | X | ─ |
| *Glenodinium gymnodinium* Penard * | X | ─ | ─ | X |
| *Gonyaulax diegensis* Kofoid | X | ─ | ─ | ─ |
| *Gonyaulax gracilis* Schiller * | ─ | ─ | ─ | X |
| *Gonyaulax minuta* Kofoid & Michener | X | X | X | X |
| *Gonyaulax polygramma* Stein | ─ | X | X | ─ |
| *Gonyaulax spinifera* (Claparède & Lachmann) Diesing ● | X | X | X | X |
| *Gonyaulax turbynei* Murray & Whitting | X | ─ | X | ─ |
| *Gymnodinium aureolum* (E.M.Hulburt) Gert Hansen * | X | ─ | ─ | ─ |
| *Gymnodinium catenatum* H.W.Graham * ● | X | ─ | ─ | ─ |
| *Gymnodinium paradoxum* A.J.Schilling * | X | ─ | ─ | ─ |
| *Gyrodinium estuariale* E.M.Hulbert * | ─ | ─ | X | ─ |
| *Gyrodinium glaciale* Hada * | ─ | ─ | ─ | X |
| *Gyrodinium lachryma* (Meunier) Kofoid & Swezy * | ─ | ─ | ─ | X |
| *Heterocapsa rotundata* (Lohmann) G.Hansen * | X | X | ─ | ─ |
| *Histoneis depressa* Schiller | X | ─ | ─ | ─ |
| *Karenia digitata* Z.B.Yang, H.Takayama, K.Matsuoka & I.J.Hodgkiss * ● | ─ | ─ | X | ─ |
| *Karenia mikimotoi* (Miyake & Kominami ex Oda) Gert Hansen & Ø.Moestrup ● | ─ | X | X | ─ |
| *Karlodinium conicum* Salas * ● | X | ─ | ─ | ─ |
| *Karlodinium micrum* (B.Leadbeater & J.D.Dodge) J.Larsen * | X | ─ | ─ | ─ |
| *Karlodinium veneficum* (D.Ballantine) J.Larsen * ● | X | ─ | ─ | ─ |
| *Kofoidinium splendens* J.Cachon & M.Cachon * | X | ─ | ─ | ─ |
| *Kofoidinium velleloides* Pavillard * | X | ─ | ─ | ─ |
| *Lebouridinium glaucum* (M.Lebour) F.Gómez, H.Takayam, D.Moreira & P.López-García * | X | ─ | ─ | X |
| *Lingulodinium polyedra* (F.Stein) J.D.Dodge ● | ─ | X | X | ─ |
| *Ornithocercus steinii* Schütt | X | ─ | ─ | ─ |
| *Ostreopsis ovata* Fukuyo ● | ─ | ─ | X | ─ |
| *Oxytoxum laticeps* Schiller | X | X | ─ | ─ |
| *Oxytoxum longiceps* Schiller | X | ─ | X | ─ |
| *Oxytoxum sceptrum* (F.Stein) Schröder | X | ─ | ─ | ─ |
| *Oxytoxum sphaeroideum* Stein | ─ | ─ | X | ─ |
| *Phalacroma oxytoxoides* (Kofoid) F.Gomez, P.Lopez-Garcia & D.Moreira * | X | X | X | X |
| *Phalacroma rotundatum* (Claparéde & Lachmann) Kofoid & Michener ● | ─ | ─ | X | X |
| *Podolampas palmipes* Stein | ─ | X | X | X |
| *Podolampas spinifera* Okamura | ─ | ─ | ─ | X |
| *Polykrikos* sp. * | ─ | X | ─ | ─ |
| *Prorocentrum* sp. | X | ─ | ─ | ─ |
| *Pronoctiluca pelagica* Fabre-Domergue * | X | ─ | X | ─ |
| *Prorocentrum balticum* (Lohmann) Loeblich * | X | X | X | X |
| *Prorocentrum compressum* (Bailey) Abé ex J.D.Dodge | X | X | X | X |
| *Prorocentrum dentatum* Stein * | ─ | X | ─ | X |
| *Prorocentrum gracile* Schütt ● | X | X | X | X |
| *Prorocentrum mexicanum* Osorio-Tafall ● | X | X | X | X |
| *Prorocentrum micans* Ehrenberg | X | X | ─ | X |
| *Prorocentrum minimum* (Pavillard) J.Schiller ● | X | X | ─ | X |
| *Prorocentrum ovum* (Schiller) J.D.Dodg * | ─ | ─ | X | ─ |
| *Prorocentrum scutellum* Schröder | ─ | ─ | X | ─ |
| *Prorocentrum triestinum* J.Schiller * | X | X | X | ─ |
| *Protoperidinium achromaticum* (Levander) Balech * | ─ | ─ | X | ─ |
| *Protoperidinium biconicum* (P.-A.Dangeard) Balech * | X | X | ─ | X |
| *Protoperidinium brevipes* (Paulsen) Balech | X | X | X | X |
| *Protoperidinium brochii* (Kofoid & Swezy) Balech | ─ | X | ─ | ─ |
| *Protoperidinium cerasus* (Paulsen) Balech | X | X | X | X |
| *Protoperidinium claudicans* (Paulsen) Balech | X | X | ─ | X |
| *Protoperidinium conicoides* (Paulsen) Balech * | ─ | ─ | X | ─ |
| *Protoperidinium conicum* (Gran) Balech | X | X | X | X |
| *Protoperidinium crassipes* (Kofoid) Balech | X | ─ | ─ | X |
| *Protoperidinium curvipes* (Ostenfeld) Balech | ─ | ─ | ─ | X |
| *Protoperidinium depressum* (Bailey) Balech | X | X | X | X |
| *Protoperidinium divergens* (Ehrenberg) Balech | X | X | X | X |
| *Protoperidinium furcatum* (Abé) Balech * | ─ | ─ | X | ─ |
| *Protoperidinium globulus* (F.Stein) Balech | X | ─ | X | X |
| *Protoperidinium inflatum* (Okamura) Balech | ─ | X | ─ | ─ |
| *Protoperidinium latispinum* (Mangin) Balech * | ─ | X | ─ | ─ |
| *Protoperidinium latissimum* (Kofoid) Balech * | ─ | ─ | ─ | X |
| *Protoperidinium marielebouriae* (Paulsen) Balech * | X | ─ | ─ | ─ |
| *Protoperidinium minutum* (Kofoid) Loeblich | ─ | X | ─ | ─ |
| *Protoperidinium obliquum* Dangeard * | X | ─ | ─ | ─ |
| *Protoperidinium oblongum* (Aurivillius) Parke & Dodge | X | X | X | X |
| *Protoperidinium ovatum* Pouchet | X | X | X | X |
| *Protoperidinium pacificum*(Kofoid & Michener) F.J.R.Taylor & Balech ex Balech * | ─ | ─ | X | ─ |
| *Protoperidinium pellucidum* Bergh | X | X | ─ | ─ |
| *Protoperidinium persicum* Schiller * | X | ─ | ─ | ─ |
| *Protoperidinium punctulatum* (Paulsen) Balech | X | ─ | ─ | ─ |
| *Protoperidinium pyriforme* (Paulsen) Balech * | X | X | X | X |
| *Protoperidinium quarnerense* (B.Schröder) Balech | ─ | ─ | ─ | X |
| *Protoperidinium* sp. | X | ─ | ─ | X |
| *Protoperidinium spirale* (Gaarder) Balech * | X | X | X | ─ |
| *Protoperidinium steinii* (Jørgensen) Balech | X | X | X | X |
| *Protoperidinium subsphaericum* (Broch) Balech | ─ | X | X | X |
| *Protoperidinium tenuissimum* (Kofoid) Balech * | ─ | X | ─ | ─ |
| *Protoperidinium thorianum*(Paulsen) Balech * | ─ | ─ | X | ─ |
| *Pseudophalacroma nasutum* Jorgensen * | ─ | ─ | ─ | X |
| *Pyrophacus horologium* F.Stein | X | X | X | X |
| *Scrippsiella trochoidea* (Stein) Loeblich III ● | X | X | X | X |
| *Triadinium sphaericum* (Murray & Whitting) Dodge | X | ─ | ─ | ─ |
| *Tripos brevis* (Ostenf. & Johannes Schmidt 1901) F. Gómez, comb. nov. | X | ─ | ─ | ─ |
| *Tripos brevis* var. *curvulus* (Jørgesen) F.Gómez | X | X | X | ─ |
| *Tripos californiensis* (Kofoid) F.Gómez * | X | X | X | ─ |
| *Tripos candelabrus* f. *depressus* (Pouchet) F.Gómez | X | ─ | ─ | ─ |
| *Tripos candelabrus* (Ehrenberg) F.Gómez | X | ─ | X | ─ |
| *Tripos carriensis* (Gourret) F.Gómez | X | ─ | X | X |
| *Tripos contortus* (Gourret) F.Gómez | X | ─ | X | ─ |
| *Tripos declinatus* f. *normalis* (Jørgesen) F.Gómez | X | ─ | X | ─ |
| *Tripos dens* (Ostenfeld & Johannes Schmidt) F.Gómez | X | ─ | ─ | ─ |
| *Tripos egyptiacus* (Halim) F.Gómez | X | ─ | ─ | ─ |
| *Tripos extensus* (Gourret) F.Gómez | X | X | ─ | ─ |
| *Tripos falcatus* (Kofoid) F.Gómez | X | X | X | ─ |
| *Tripos furca* var*. berghii* (Lemmerm.) F.Gómez | X | ─ | ─ | ─ |
| *Tripos furca* var*. brevicornis (Lemmerm.) F. Gómez ** | ─ | ─ | X | ─ |
| *Tripos furca* var. *eugrammus* (Ehrenberg) F.Gómez | X | X | X | X |
| *Tripos furca* var. *furca* (Ehrenberg) F.Gómez ● | X | ─ | X | ─ |
| *Tripos fusus* (Ehrenberg) F.Gómez ● | X | X | X | X |
| *Tripos horridus* f. *inclinatus* (Kofoid) F.Gómez | X | ─ | ─ | X |
| *Tripos horridus* var. *buceros* (O. Zacharias) F. Gómez | X | X | X | X |
| *Tripos kofoidii* (Jörgenen) F.Gómez | X | ─ | X | X |
| *Tripos macroceros* (Ehrenberg) F.Gómez | X | ─ | ─ | ─ |
| *Tripos massiliensis* var. *macroceroides* (G. Karst.) F. Gómez | X | ─ | X | ─ |
| *Tripos muelleri* f. *parallelus*(Schmidt) F.Gómez | X | ─ | ─ | ─ |
| *Tripos semipulchellus* (Jørgesen) F.Gómez | X | ─ | ─ | ─ |
| *Tripos teres* (Kofoid) F.Gómez | X | ─ | X | ─ |
| *Tripos trichoceros* (Ehrenb.) F. Gómez | X | X | X | X |
| *Zygabikodinium lenticulatum* Loeblich Jr. & Loeblich III | ─ | ─ | X | ─ |
| **Cyanobacteria** |  |  |  |  |
| *Cephalothrix komarekiana* C.F.S.Malone *et al. ** | X | ─ | ─ | ─ |
| *Chroococcus minutus* (Kützing) Nägeli | ─ | X | ─ | ─ |
| *Chroococcus polyhedriformis* Schmidle * | ─ | X | ─ | ─ |
| *Chroococcus turgidus* (Kützing) Nägeli | X | X | X | ─ |
| *Cylindrospermopsis curvispora* M.Watanabe * | ─ | X | ─ | ─ |
| *Cylindrospermopsis raciborskii* (Woloszynska) Seenayya & Subba Raju * ● | X | ─ | ─ | ─ |
| *Glaucospira laxissima* (G.S.West) Simic, Komárek & Dordevic * | ─ | ─ | X | ─ |
| *Johannesbaptistia pellucida* (Dickie) W.R.Taylor & Drouet * | X | ─ | ─ | ─ |
| *Komvophoron minutum* (Skuja) Anagnostidis & Komárek * | X | X | X | X |
| *Leptolyngbya* sp. | ─ | ─ | ─ | X |
| *Merismopedia minima* G.Beck * | ─ | ─ | X | ─ |
| *Oscillatoria formosa* Bory ex Gomont | ─ | ─ | ─ | X |
| *Oscillatoria limosa* C.Agardh ex Gomont | X | X | X | X |
| *Oscillatoria margaritifera* Kützing ex Gomont * | X | ─ | X | ─ |
| *Oscillatoria princeps* Vaucher ex Gomont * | X | ─ | ─ | ─ |
| *Oscillatoria putrida* Schmidle * | X | ─ | ─ | X |
| *Oscillatoria sancta* Kützing ex Gomont | X | ─ | ─ | ─ |
| *Oscillatoria simplicissima* Gomont | X | X | X | ─ |
| *Oscillatoria* sp. | ─ | X | ─ | ─ |
| *Oscillatoria tenuis* C.Agardh ex Gomont | ─ | ─ | ─ | X |
| *Phormidium formosum* (Bory ex Gomont) Anagnostidis & Komárek | ─ | ─ | ─ | X |
| *Phormidium* sp.1 | ─ | ─ | X | X |
| *Phormidium* sp.2 | ─ | ─ | X | ─ |
| *Planktothrix agardhii* (Gomont) Anagnostidis & Komárek | X | ─ | X | ─ |
| *Pseudanabaena catenata* Lauterborn * | X | ─ | ─ | ─ |
| *Pseudophormidium tenue* (Thuret ex Gomont) Anagnostidis & Komárek * | ─ | X | ─ | ─ |
| *Richelia intracellularis* J.A.Schmidt | X | ─ | ─ | ─ |
| *Scytonema* sp. * | X | ─ | ─ | ─ |
| *Sphaerocystis planctonica* (Korshikov) Bourrelly * | ─ | ─ | ─ | X |
| *Stigonema* sp. * | X | ─ | ─ | ─ |
| *Trichodesmium* cf. *lacustre* Klebahn | X | ─ | ─ | ─ |
| *Trichodesmium* cf. *thiebautii* Gomont ex Gomont | ─ | ─ | X | ─ |
| *Wolskyella australis* G.McGregor & J.Kaštovský * | ─ | X | ─ | ─ |
| **Chlorophytes** |  |  |  |  |
| *Actinastrum hantzschii* Lagerheim | X | ─ | ─ | ─ |
| *Chlorella* sp. | X | X | ─ | X |
| *Closteriopsis longissima* (Lemmermann) Lemmermann * | X | ─ | ─ | ─ |
| *Closterium acerosum* Ehrenberg ex Ralfs * | ─ | X | ─ | X |
| *Closterium gracile* Brébisson ex Ralfs | ─ | ─ | ─ | X |
| *Cosmarium* cf. *obsoletum* (Hantzsch) Reinsch * | ─ | ─ | ─ | X |
| *Cosmarium contractum* var. *minutum*(Delponte) Coesel * | ─ | ─ | ─ | X |
| *Pediastrum biwae* Negoro | X | ─ | X | ─ |
| *Pediastrum boryanum* (Turpin) Meneghini * | X | ─ | ─ | ─ |
| *Pediastrum duplex* Meyen | ─ | ─ | X | ─ |
| *Pediastrum simplex* Meyen | X | X | X | ─ |
| *Pyramimonas* sp. Schmarda * | X | ─ | X | ─ |
| *Scenedesmus armatus* (Chodat) Chodat * | ─ | X | ─ | ─ |
| *Scenedesmus dimorphus* (Turpin) Kützing | ─ | ─ | X | ─ |
| *Scenedesmus longispina* Chodat * | X | ─ | ─ | ─ |
| *Scenedesmus obtusus* Meyen * | ─ | X | ─ | ─ |
| *Scenedesmus quadricauda* (Turpin) Brébisson | ─ | X | ─ | ─ |
| *Staurastrum gracile* Ralfs ex Ralfs | X | ─ | ─ | ─ |
| *Pterosperma* cf. *polygonum* Ostenfeld | ─ | X | ─ | ─ |
| *Tetrastrum* sp. Chodat * | ─ | ─ | ─ | X |
| **Euglenophytes** |  |  |  |  |
| *Astasia klebsii* Lemmermann * | ─ | X | ─ | ─ |
| *Euglena deses* Ehrenberg * | X | ─ | ─ | X |
| *Euglena gracilis* G.A.Klebs | X | X | ─ | ─ |
| *Euglena proxima* P.A.Dangeard * | ─ | X | ─ | ─ |
| *Euglena* sp. | X | ─ | ─ | ─ |
| *Euglena spirogyra* Ehrenberg * | ─ | ─ | X | ─ |
| *Euglena viridis* (O.F.Müller) Ehrenberg * | X | X | X | ─ |
| *Eutreptiella braarudii* Throndsen * | X | X | X | ─ |
| *Lepocinclis salina* F.E.Fritsch * | X | ─ | ─ | ─ |
| *Trachelomona*s sp. * | ─ | ─ | ─ | X |
| **Raphidophytes** |  |  |  |  |
| *Chattonella* cf. *marina* (Subrahmanyan) Y.Hara & M.Chihara * ● | ─ | ─ | ─ | X |
| *Chattonella* cf. *subsalsa* B.Biecheler * ● | X | ─ | X | ─ |
| **Eustigmatophytes** |  |  |  |  |
| *Nannochloropsis* sp. * | X | ─ | ─ | X |
| **Charophytes** |  |  |  |  |
| *Mougeotia parvula* Hassall * | ─ | ─ | ─ | X |
| **Silicoflagellates** |  |  |  |  |
| *Dictyocha fibula* Ehrenberg ● | ─ | X | X | X |
| **Haptophyta (Coccolithophorids)** |  |  |  |  |
| *Anacanthoica cidaris* (Lecal-Schlauder) Kleijne 1992 * | X | ─ | ─ | ─ |
| *Calciosolenia murrayi* Gran * | ─ | ─ | ─ | X |
| *Hymenomonas* sp. | ─ | ─ | X | ─ |
| **Ebriids** |  |  |  |  |
| *Hermesinum adriaticum* O. Zacharias * | ─ | ─ | X | ─ |

Based on seasonal average abundance; V, very abundant (81–100%); A, abundant (61–80%); C, common (41–60%); R, rare (21–40%); X, present sporadically (1–20%). ● Harmful algal species; * Potentially newly introduced species in the Red Sea.

**Table 4:** Seasonal and regional variations of total phytoplankton density (cells l^-1^) and chlorophyll *a* concentration (µg. l^-1^) in Suez Bay.

**Table 5:** Seasonal and regional variations of phytoplankton diversity in Suez Bay.

**Table 6:** Statistically significant values of Pearson correlation between community properties and environmental parameters in Suez Bay (p ≤ 0.05).

**Table 7:** Correlation coefficient between phytoplankton stability (TSI), chlorophyll *a* concentration and standard deviation with environmental parameters (A), and with standard deviation of environmental parameters (B).

 Values in bold: with significance at 0.05 levels.
